# Synchronous retreat of Thwaites and Pine Island glaciers in response to external forcings in the presatellite era

**DOI:** 10.1073/pnas.2211711120

**Published:** 2024-02-26

**Authors:** Rachel W. Clark, Julia S. Wellner, Claus-Dieter Hillenbrand, Rebecca L. Totten, James A. Smith, Lauren E. Miller, Robert D. Larter, Kelly A. Hogan, Alastair G. C. Graham, Frank O. Nitsche, Asmara A. Lehrmann, Allison P. Lepp, James D. Kirkham, Victoria T. Fitzgerald, Georgina Garcia-Barrera, Werner Ehrmann, Lukas Wacker

**Affiliations:** ^a^Department of Earth and Atmospheric Sciences, University of Houston, Houston, TX 77004; ^b^British Antarctic Survey, Cambridge CB3 0ET, United Kingdom; ^c^Department of Geological Sciences, University of Alabama, Tuscaloosa, AL 35401; ^d^Department of Environmental Sciences, University of Virginia, Charlottesville, VA 22903; ^e^College of Marine Science, University of South Florida, St. Petersburg, FL 33701; ^f^Lamont-Doherty Earth Observatory of Columbia University, New York, NY 10964; ^g^Scott Polar Research Institute, University of Cambridge, Cambridge CB2 1ER, United Kingdom; ^h^Institute for Geophysics & Geology, University of Leipzig, Leipzig 04103, Germany; ^i^Ion Beam Physics, Eidgenössische Technische Hochschule Zürich, Zürich 8093, Switzerland

**Keywords:** Antarctica, sedimentology, glacial marine geology, glacial history, sea level

## Abstract

Today, relatively warm Circumpolar Deep Water is melting Thwaites Glacier at the base of its ice shelf and at the grounding zone, contributing to significant ice retreat. Accelerating ice loss has been observed since the 1970s; however, it is unclear when this phase of significant melting initiated. We analyzed the marine sedimentary record to reconstruct Thwaites Glacier’s history from the early Holocene to present. Marine geophysical surveys were carried out along the floating ice-shelf margin to identify core locations from various geomorphic settings. We use sedimentological data and physical properties to define sedimentary facies at seven core sites. Glaciomarine sediment deposits reveal that the grounded ice in the Amundsen Sea Embayment had already retreated to within ~45 km of the modern grounding zone prior to ca. 9,400 y ago. Sediments deposited within the past 100+ y record abrupt changes in environmental conditions. On seafloor highs, these shifts document ice-shelf thinning initiating at least as early as the 1940s. Sediments recovered from deep basins reflect a transition from ice proximal to slightly more distal conditions, suggesting ongoing grounding-zone retreat since the 1950s. The timing of ice-shelf unpinning from the seafloor for Thwaites Glacier coincides with similar records from neighboring Pine Island Glacier. Our work provides robust new evidence that glacier retreat in the Amundsen Sea was initiated in the mid-twentieth century, likely associated with climate variability.

## Significance

Thwaites Glacier plays a vital role in regulating West Antarctic Ice Sheet stability and, thus, global sea-level rise. Marine sediments seaward of the glacier reveal that the grounding zone had retreated to its current position before 9,400 y ago. The floating ice shelf fringing Thwaites Glacier lost contact with seafloor highs in the mid-twentieth century, simultaneously with the ice shelf at neighboring Pine Island Glacier. The synchronous ice retreat of these two major ice streams suggests that, rather than being driven by internal dynamics unique to each glacier, retreat in the Amundsen Sea drainage sector results from external oceanographic and atmospheric drivers, which recent modelling studies show are modulated by climate variability.

Recent numerical simulations estimate that the Antarctic contribution to global mean sea-level rise, which is predominantly caused by West Antarctic Ice Sheet (WAIS) melting, will reach approximately 5 to 6 cm by the year 2,100 Common Era (C.E.) and between ca. 2.5 to 2.9 m by the year 2,500 C.E. (1). However, estimates vary widely (2) due to lack of detailed understanding of how ice sheets will respond to ongoing climate fluctuations. Thwaites Glacier is a key ice stream draining the WAIS into the eastern Amundsen Sea Embayment, accounting for four percent of present-day sea-level rise from ca. 1993 to 2017 C.E. (3–5). Its catchment extends far into the ice sheet interior on an inland-deepening bed. Therefore, ice loss from Thwaites Glacier could eventually compromise the stability of the entire WAIS and promote increased and accelerated contributions to sea-level rise in the future (6).

The WAIS is a marine-based and marine-terminating ice sheet, meaning that its bed lies mostly below sea level and that it discharges ice into the ocean either directly or, more commonly, via floating ice shelves, which makes it susceptible to oceanographic influences. Today, warm Circumpolar Deep Water (CDW) is flowing onto the Amundsen Sea continental shelf and melting glaciers, such as Thwaites Glacier, at their grounding zones and the undersides of their ice shelves (7, 8). As a result of this melting and the dynamic response of upstream ice, the floating and grounded parts of Thwaites Glacier are currently thinning, the ice stream’s grounding zone is retreating, and its flow speed is accelerating (9–11). These trends were identified mainly through airborne radar and satellite-derived observations extending to the late 1970s when ice loss was already underway (5, 12, 13). Thus, it remains unknown when the current phase of glacial retreat began at Thwaites Glacier.

**Fig. 1.**
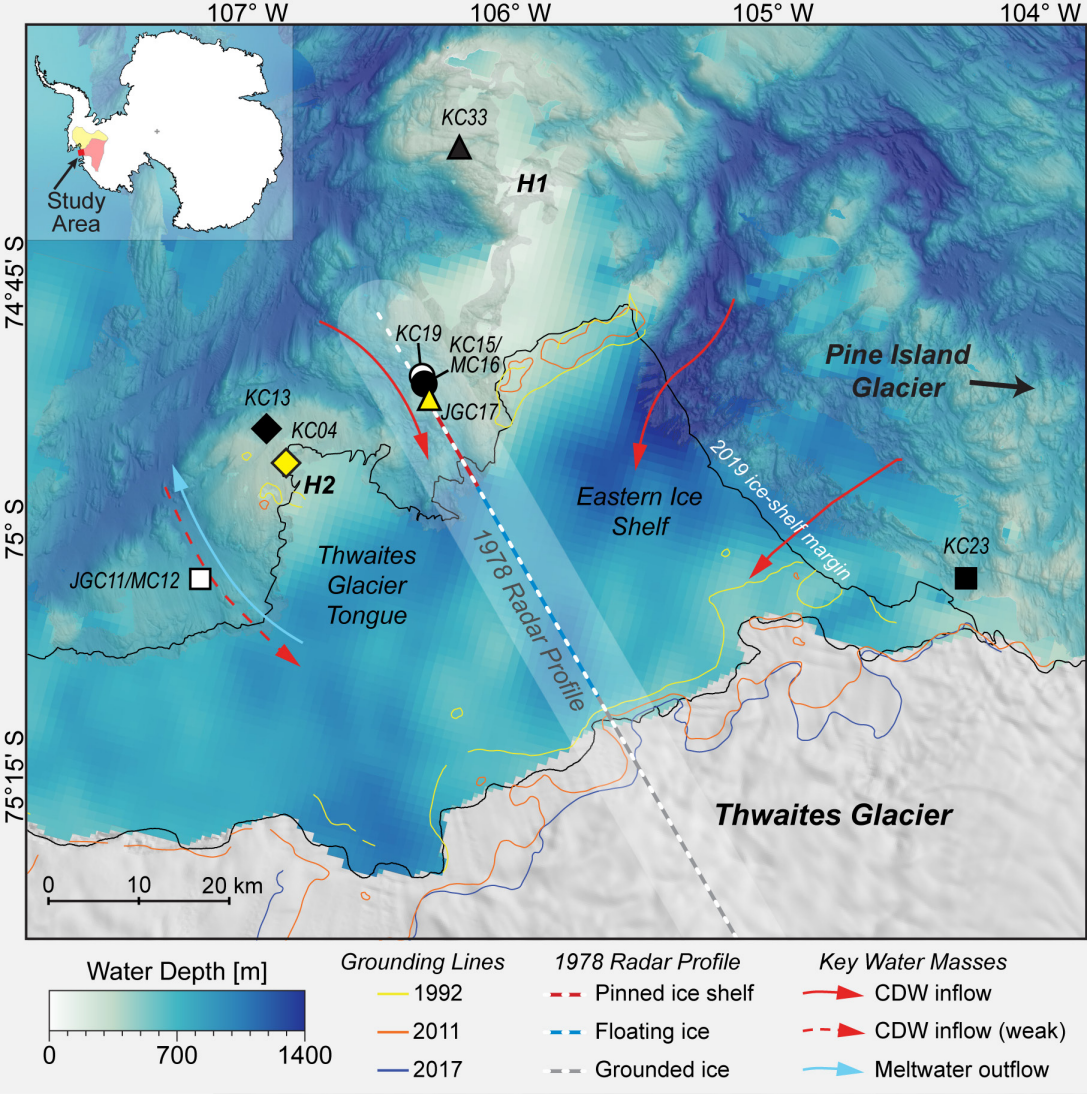
Bathymetric map and core locations around the Thwaites Glacier margin. Inset shows location of detailed map (red) and the extent of Thwaites Glacier (pale red) and Pine Island Glacier (pale yellow) catchments. Multibeam bathymetry is compiled from NBP19-02 shipboard data and previous work (15, 30). Lower resolution, satellite-derived bathymetry is shown for sub-ice shelf areas and in places without multibeam bathymetry (31). Two major seafloor highs (H1 and H2) are indicated. REMA digital elevation model shown over grounded ice (32). Approximate grounding-zone locations are shown with lines for years: 1992 C.E. and 2011 C.E. (33); 2017 C.E. (34). Ice-shelf calving margin is from March 2019 C.E. (15). Location of an airborne radar profile collected in 1978 C.E. (dashed line) is also shown with a 10 km wide uncertainty window (13). New core data from NBP19-02 KC04 to KC23 discussed in text. Previous work on cores NBP19-02 KC04, KC23, and NBP20-02 KC33 mentioned in text (25). Red arrows mark CDW flow under the ice shelf, and the light blue arrow illustrates meltwater-enriched outflow (7).

To investigate when the modern phase of retreat at Thwaites Glacier initiated, we collected marine sediment cores from a range of water depths along the calving front. Thwaites Glacier’s floating margin is composed of the Eastern Ice Shelf, which remains pinned to (resting on) a seafloor high, and the Thwaites Glacier Tongue, which lost contact with the seafloor in 2011 (Fig. 1) (10, 14). The seafloor around Thwaites Glacier is high relief, recording patterns of past ice flow and grounding-zone retreat (15). Marine sediment records indicate that the ice sheet extended to the continental shelf break during the Last Glacial Maximum (ca. 19 to 23 calibrated thousand years (cal. ka B.P.) with respect to 1950 C.E.); subsequently, grounded ice retreated into the inner continental shelf and reached a configuration similar to present-day ice margins by 10 cal. ka BP (16–20). Relative sea-level reconstructions indicate that the grounded ice did not experience significant retreat and readvance during the mid-Holocene to present (21). Marine sediment cores in this region, described in previous work, reveal a variety of sediment types, i.e., facies, such as glaciomarine mud, glaciomarine diamicton (a poorly sorted sediment with clay- to gravel-sized grains), and subglacial till (18, 19, 22, 23). Meltwater plume deposits have been reported in this region (24, 25) and are consistent with earlier interpretations of meltwater-related geomorphology (26–29), indicating subglacial meltwater flow as an important mechanism to transport subglacially eroded detritus into the ocean beyond Thwaites Glacier. Applying a multiproxy approach to the lithologically diverse sediment record around Thwaites Glacier, we document recent ice-shelf behavior and the onset of grounded ice retreat.

## Results

Sediment cores were collected aboard the RV/IB *Nathaniel B. Palmer* during the NBP19-02 cruise (in the 2019 austral summer) using a Kasten core (KC) and jumbo gravity core (JGC) to recover sedimentary sequences up to 3 m and 6 m long, respectively, and using a megacorer (MC) to retrieve up to 0.5 m of undisturbed seafloor sediments. The core sites range in water depth from 450 to 750 m along the Thwaites Glacier margin (Fig. 1 and *SI Appendix*, Table S1). Cores JGC11/MC12 were recovered as a pair from a small trough on the western side of Thwaites Glacier Tongue. Cores KC04 and KC13 were collected from shallow sites close to the northern margin of Thwaites Glacier Tongue. The shallower site of core JGC17 is located close to the ice-shelf front between Thwaites Glacier Tongue and the Eastern Ice Shelf, while cores KC15/MC16 and KC19 were recovered from the same area but in slightly deeper water further from the modern ice front. Core KC23 was retrieved from the eastern margin of the Eastern Ice Shelf and is the site most proximal to grounded ice today.

**Fig. 2.**
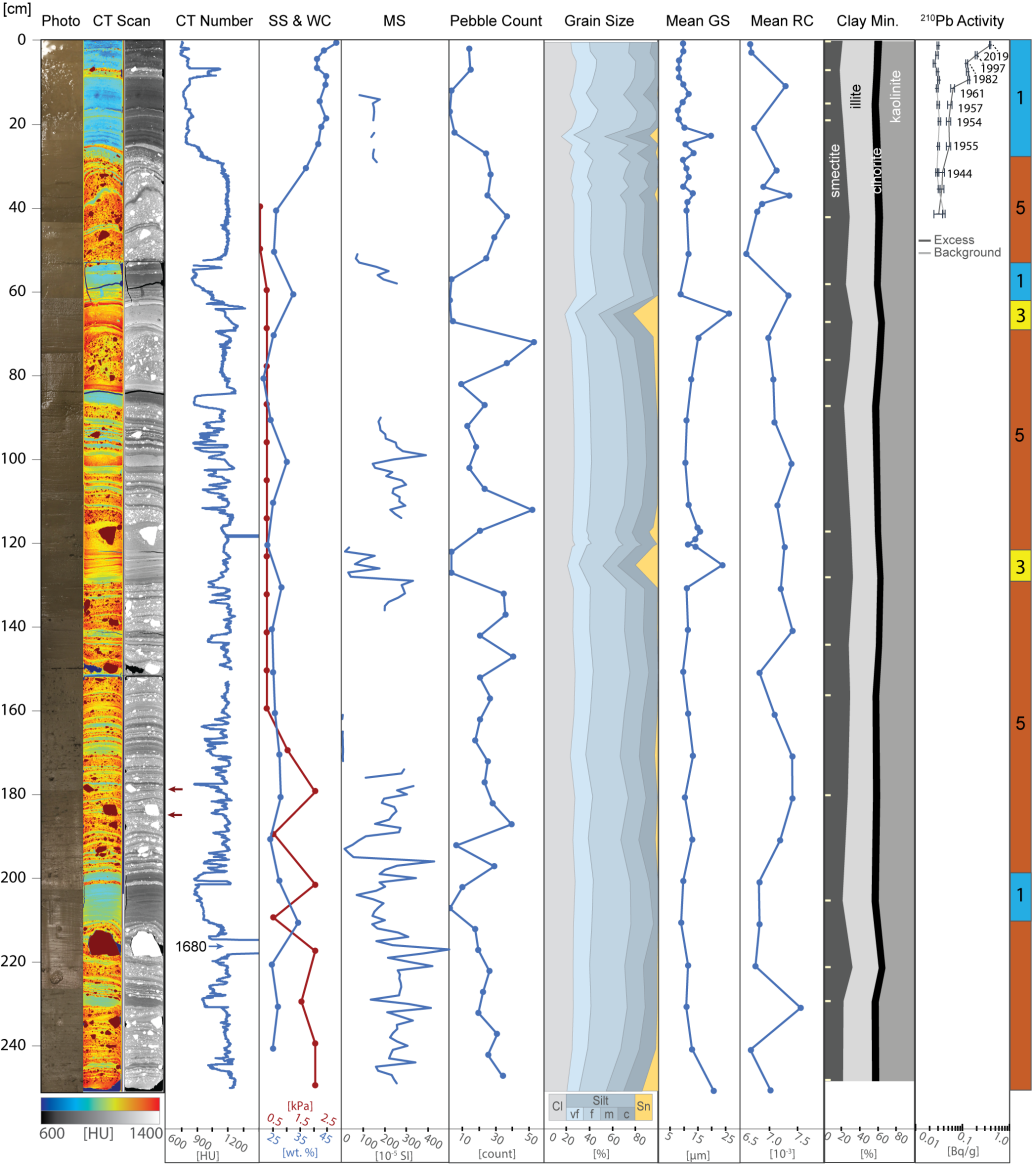
Compilation of proxies for core KC04. From left to right: line-scan core image, CT scan images in false color and grayscale (red arrows highlight depressions underneath gravel clasts), CT number, shear strength (SS) and water content (WC), point sensor magnetic susceptibility (MS), pebble count, grain size distribution, mean grain size (GS), mean sand grain roughness coefficient (RC), clay mineral assemblage (sample depths indicated with tick marks), ^210^Pb activity, and facies assignments, which are defined in Table 1. Point sensor MS was not measured where the opened core surface was uneven. Error bars are shown for individual background and excess ^210^Pb activity measurements.

**Fig. 3.**
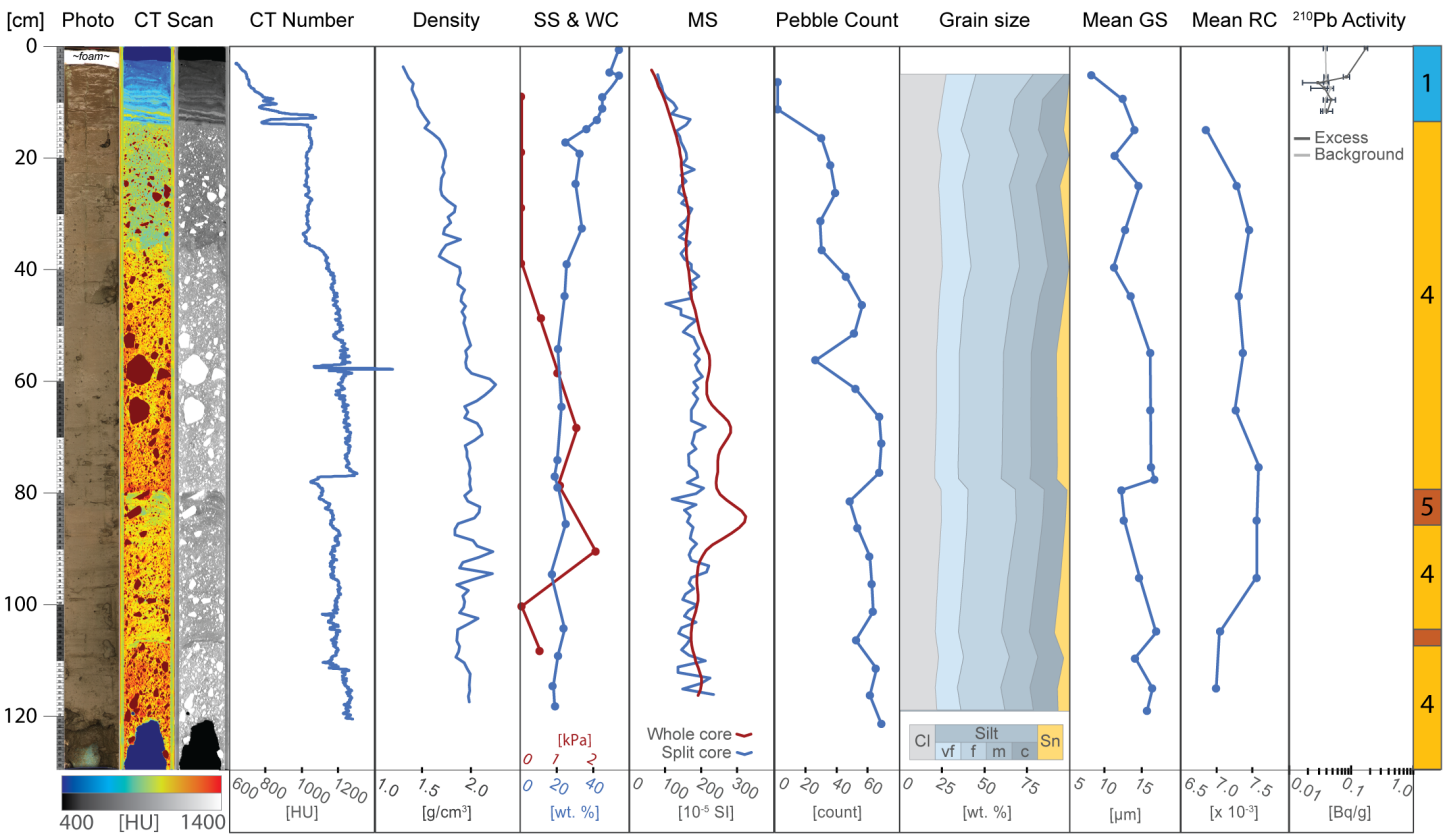
Summary of proxy data for core JGC17. From left to right: line-scan core image, CT scan images in false color and grayscale, CT number, density, shear strength (SS) and water content (WC), point-sensor and whole-core magnetic susceptibility (MS), pebble count, grain size distribution, mean grain size (GS), mean sand grain roughness coefficient (RC), ^210^Pb activity, and facies assignments, which are defined in Table 1. Error bars are shown for individual background and excess ^210^Pb activity measurements

**Fig. 4.**
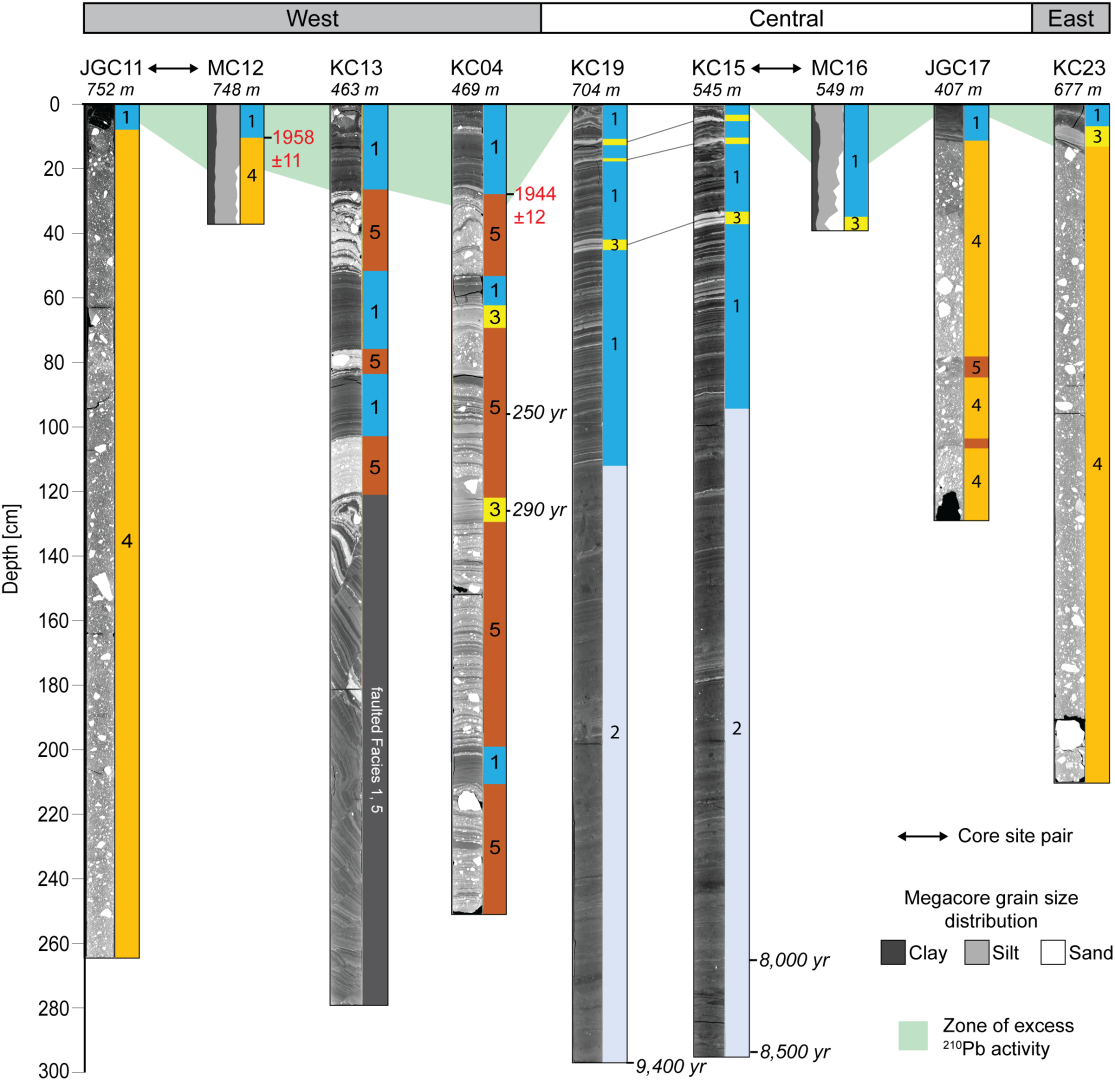
Gray scale CT scan images and facies assignments of cores collected along the Thwaites Glacier margin from west (*Left*) to east (*Right*). Water depths are given in italics below the core IDs. Grain size data for megacores MC12 (= site JGC11) and MC16 (= site KC15) are included. The pale green area marks the core intervals with excess ^210^Pb activity; no excess activity was detected in cores KC15 and KC19. Red text indicates ^210^Pb ages (in years C.E.) for the basal boundary of Facies 1 in cores KC04 and MC12; uncertainty was calculated from the SE of the ages. MICADAS ^14^C ages (in cal. yr B.P., respectively) are shown with italicized text for cores KC04, KC15, and KC19. Fine gray lines highlight Facies 3 beds that can be correlated between cores KC15 and KC19. Sediments in the lower half of KC13 are faulted as result of natural processes or coring disturbance; therefore, no facies are assigned. Compilations of all core data for JGC11, KC13, KC15, KC19, and KC23 are provided in the *SI Appendix*, Figs. S1–S5.

Sedimentological data, clay mineral provenance, and physical properties, such as water content, shear strength, magnetic susceptibility, wet bulk density, and computed tomography (CT) number (a proxy for sediment density) were used to build a facies classification scheme comprising five distinct sediment types (Table 1 and *SI Appendix*, Table S3). Core photos, X-ray images obtained by CT-scanning, analyzed parameters, and facies assignments for individual cores are provided in Figs. 2 and 3 and *SI Appendix*, Figs. S1–S6. Both ^210^Pb chronology on bulk sediment and ^14^C ages from calcareous foraminifera establish when sediments accumulated in the Holocene (Fig. 4 and *SI Appendix*, Table S2). We identify five sedimentary facies that are described in detail in the *SI Appendix* and in brief here.

**Table 1.** Summary of proxy data for sediment Facies 1 to 5

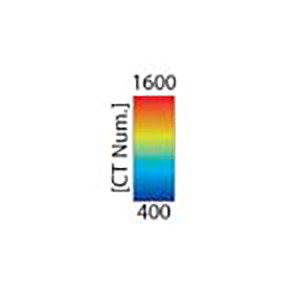	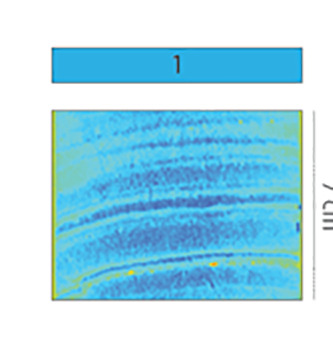	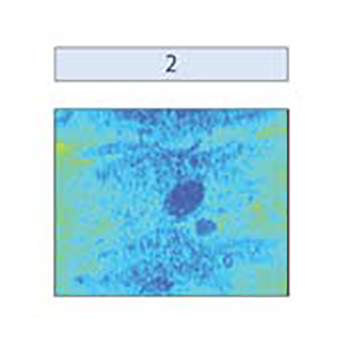	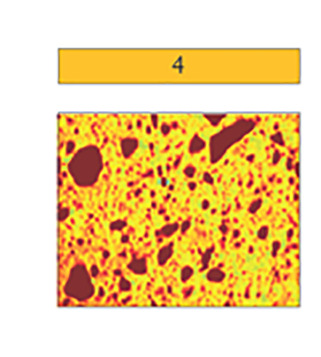	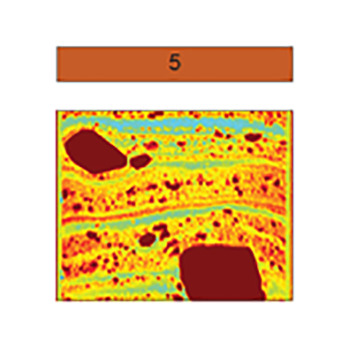	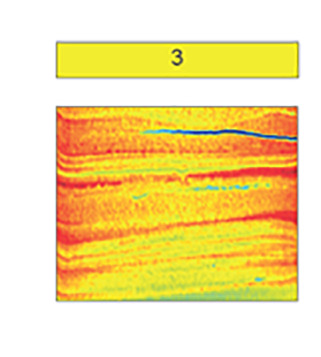
	Laminated clayey silt with sandy lenses and isolated gravel	Mottled to weakly laminated clayey silt	Laminated sandy mud	Massive diamicton	Stratified diamicton with Facies 1,3 interbeds
Microfossils	foraminifera, diatoms	foraminifera, diatoms	foraminifera	none	foraminifera
Mean [μm]	7–11	6–8	17–48	11–45	10–21
Clay [%]	22–28	23–32	12–22	14–27	18–26
Silt [%]	72–77	68–75	18–68	66–75	65–76
Sand [%]	0–7	0–3	9–42	3–38	1–16
Pebbles [per 5 cm depth]	0–15	0–5	none	14–66	16–60
Roughness [x 1^−3^]	6.6–7.5	5.7–7.7	6.9–7.4	6.8–7.6	6.5–7.8
WC [wt. %]	>30	>35	26–37	10–25	18–27
SS [kPa]	≤2	≤3	0	≤24.5	≤2
MS [10^−5^ SI]	100–200	100–150	100–250	50–800	125–500
CT Num. [HU]	700–950	800–900	≥ 1,000	1,000–1,450	900–1,200
Setting(s)	Open marine to distal sub-ice shelf	Bioturbated open marine to distal sub-ice shelf	Open marine to distal sub-ice shelf (higher energy/closer source)	Ice proximal	Proximal to pinned ice shelf

Example CT scan images shown in color; CT number values are indicated with color bar. Abbreviations: water content (WC), shear strength (SS), and magnetic susceptibility (MS).

### Facies Interpretations.

Facies 1 is a laminated clayey silt that is pervasive along the Thwaites Glacier margin. Similar deposits were identified in previous studies on sediment cores from the Amundsen Sea Embayment, where the depositional environment was interpreted to be in a seasonally open marine and/or sub-ice shelf setting (16, 18, 19, 23, 25). Recent work, which includes detailed sedimentological and geochemical analyses of KC04 and KC23, indicates that these sediments are derived from subglacial meltwater plumes (25). Sand grain roughness values are low for the most part and show only minimal variability between the facies found in this study (Table 1). Lower roughness values, which are associated with fluvial processes would be consistent with a subglacial meltwater source and suggest the possibility of subglacial meltwater transport (35). This indicates that hydraulic transport processes (e.g., fluvial-like meltwater transport in the subglacial setting) influenced Facies 1 sediments, while higher roughness values in other facies reflect glacial abrasion of sediment grains with little to no influence from hydraulic processes (24, 36). However, the lack of variability between facies indicates that subglacial meltwater deposition is either pervasive across different depositional environments or that grain roughness is nondiagnostic of subglacial meltwater plumes. Laminations and occasional occurrences of gravel-sized clasts indicate that Facies 1 was formed most likely through (hemi-)pelagic settling of meltwater plume sediments with a minor component of ice rafting. These processes can happen both in a distal sub-ice shelf (37) or an open marine (i.e. seasonally ice free) setting (24, 25). A discrete layer of coarse-grained clasts, which we interpret as ice-rafted debris (IRD) transported and deposited either by an ice shelf or by individual icebergs, is observed at 7 to 8 cm below the seafloor in core KC04 (Fig. 2). Clay mineral analysis of the matrix of this IRD layer reveals it has a signature similar to sediments sourced from neighboring Pine Island Glacier (*SI Appendix*, Table S3) (38). This implies that the IRD layer was deposited by icebergs that calved from Pine Island Glacier in the mid- to late twentieth century (*SI Appendix*, Text) (39).

Facies 2 is a moderately bioturbated clayey silt with finer overall grain size than Facies 1 (Table 1). The origin of Facies 2 is likely not very different from that of Facies 1 (*SI Appendix*, Figs. S3 and S4). The presence of both bioturbation and benthic foraminifera in Facies 2 suggests a distal, seasonally open-marine environment, where food particles (i.e., mainly dead plankton) settle directly from the overlying surface water and provide nutrients to the benthic fauna. If this was the case, Facies 2 would represent a setting that is more distal from glacial ice than Facies 1. Facies 2 also has the largest range of grain roughness values, which is similar to roughness values measured in sub-ice shelf (described as ‘glaciomarine’) units in the Ross Sea (Table 1) (36). Grain roughness variability indicates Facies 2 has a different origin than most other facies, with detrital particles being supplied by ocean currents and ice rafting from multiple sources and, thus, probably from a larger geographic area (24, 36). A similar facies was observed by Lepp et al. (25) in the upper ca. 2.4 m of core NBP20-02 KC33, which was collected north of the Eastern Ice Shelf on the H1 bathymetric high (Fig. 1). Unlike the similar facies in KC33, the bioturbated, fine-grained Facies 2 in cores KC15 and KC19 is not present at the core surface but only below ca. 1 m core depth (*SI Appendix*, Figs. S3 and S4). Compared to other facies, clay mineral data in cores KC15 and KC19 shows lower kaolinite and higher illite content within Facies 2, which resembles the clay mineral signature of detritus supplied from Pine Island Glacier, possibly within the westward flowing coastal current (*SI Appendix*, Table S3) (37, 38, 40). Although our preferred interpretation is that Facies 2 was deposited in a seasonally open-marine setting, we cannot rule out that, alternatively, these facies may have formed in a sub-ice shelf setting relatively distal from both the grounding zone and the calving line. Such locations can only be reached by very fine-grained detritus delivered by currents from the grounding zone and/or the open ocean beyond the calving front. This depositional setting therefore resembles a ‘null zone,’ which is known to exist under large Antarctic ice shelves (41–43).

Facies 3 is a sandy mud that has similar sedimentary structures to Facies 1. However, its coarser mean grain size indicates that transport energy must have been higher to form Facies 3, or that the source of glacigenic detritus was closer to the site of deposition. Compared to Facies 1, which is pervasive in the study area, Facies 3 is less common and only reaches a maximum thickness of 10 cm in KC04 (Fig. 2), suggesting conditions which are short-lived and/or less pervasive across the Thwaites Glacier margin. Similar facies were previously described from elsewhere in the Amundsen Sea Embayment and interpreted as proximal glaciomarine facies, subject to more intense current transport and possibly even winnowing (18, 19).

**Fig. 5.**
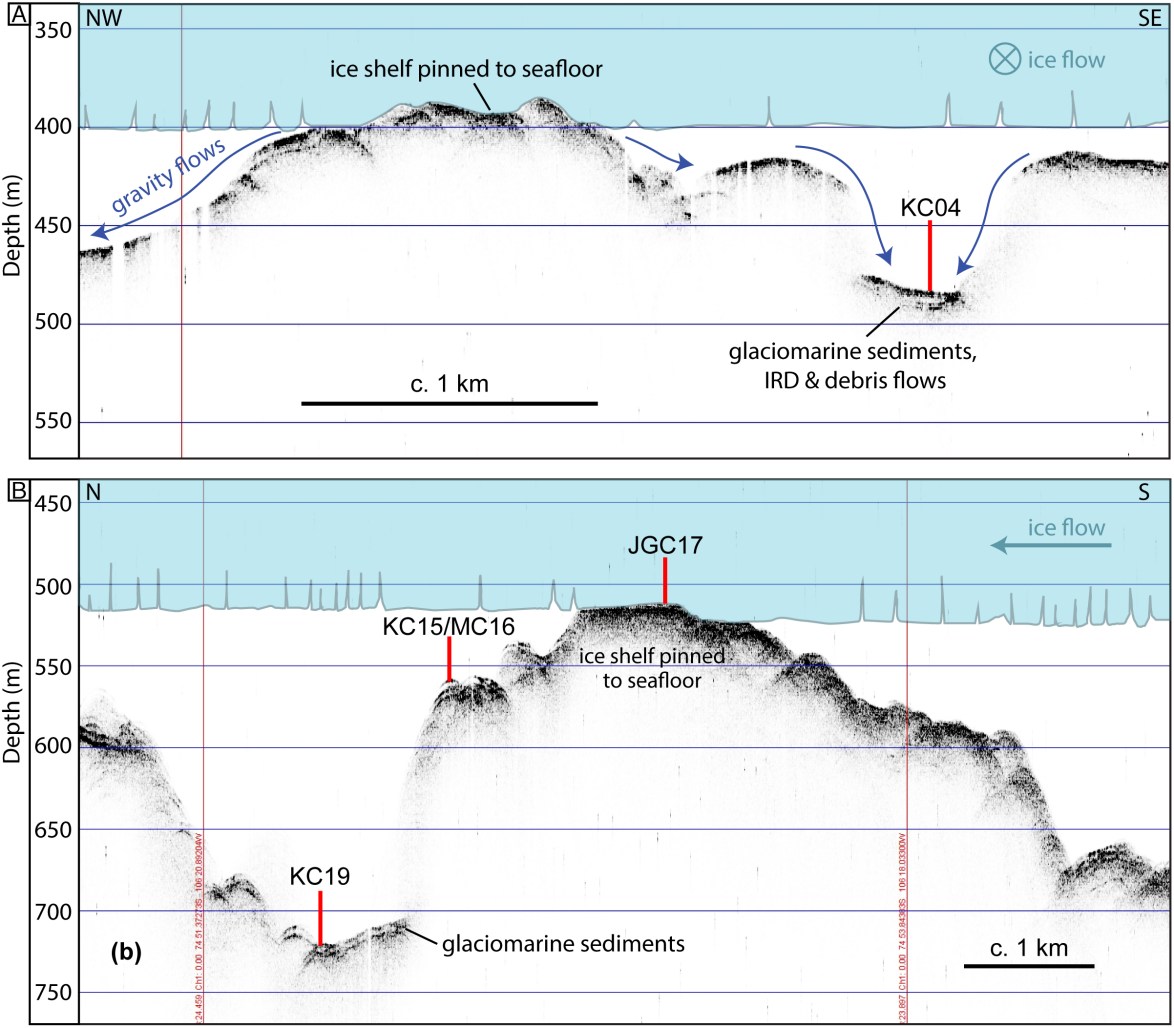
Acoustic sub-bottom profiles across seafloor highs H2 (*A*) and H1 (*B*), which are former ice-shelf pinning points. The upper image shows the location of core site KC04 within a local basin on the H2 summit. The blue area schematically illustrates past ice shelf pinning near site KC04. Dark blue arrows indicate most likely directions of gravitational sediment transport. The lower image shows the locations of core sites KC15/MC16, JGC17 and KC19 on the SW flank of H1. Depths shown here are approximate as they are derived from a simple conversion of two-way travel time to depth assuming a constant acoustic velocity of 1,500 m/s.

Facies 4, by contrast, is a clast-rich, internally structureless diamicton and was recovered at sites JGC11, JGC17, and KC23. This diamicton was likely deposited in a setting proximal to grounded ice and did not undergo significant sorting during transport. Facies 4 is characterized by low shear strength values, with the exception of the basal sediments in JGC11, where shear strength reaches up to 24.5 kPa (*SI Appendix*, Fig. S1). Such shear strength values had been reported for lithologically similar soft tills recovered elsewhere on the West Antarctic continental shelf (44–46) but also for glacigenic debris flows (45). Cores JGC11 and KC23 were obtained from water depths of 752 m and 677 m, respectively, and are the two cores located most proximal to the present-day grounding zone. Facies 4 units in these two cores show a subtle fining upward trend, suggesting that these sediments are mass flow deposits, most likely originating from the grounding zone. Our interpretation for Facies 4 in JGC17 is slightly different because JGC17 is located on a flat-topped seafloor high in 507 m water depth (Fig. 5). In this setting, the ice shelf ploughed across the seafloor, planing the seafloor high and reworking previously deposited till. Unlike JGC11 and KC23, site JGC17 has three distinct Facies 4 intervals separated by two Facies 5 intervals. These alternating packages suggest that the ice shelf had intermittent contact with the seafloor.

Facies 5 is a stratified diamicton that is observed only on the H1 and H2 submarine ridges (15). Core sites KC04, KC13, and JGC17 are located at or near previous pinning points, where the floating ice shelf had run aground on seafloor highs (Fig. 5). This former pinning at the JGC17 core site is documented in an airborne radar survey (13), while previous pinning near sites KC04 and KC13 is documented in satellite radar interferometry surveys (Fig. 1) (9, 10). Based on the geomorphic context, Facies 5 accumulated underneath the ice shelf, close to a pinning point. In this dynamic setting, the alternation between thick coarse-grained beds (mass flows) and thin fine-grained, better sorted interbeds (hemipelagic deposits) indicates that the dominant sediment transport and deposition style varied through time. In KC04, ^210^Pb and ^14^C ages indicate the upper meter of sediment, which includes alternating facies, accumulated over the past few centuries (Fig. 4 and *SI Appendix*, Table S2). A similar facies was documented in cores collected from a submarine ridge beneath the Pine Island Glacier ice shelf (37).

## Discussion

Using sedimentary facies and geochronology, the marine sediment record around Thwaites Glacier documents long-term grounding-zone stability since the early Holocene. By contrast, during the twentieth century, ice retreat and widespread meltwater plume deposition are recorded in abrupt facies transitions (i.e., Facies 1 overlies Facies 4 and 5). Previous work on sedimentary records from beneath the Pine Island Glacier ice shelf documents similar transitions and timings of ice retreat in the twentieth century, suggesting a regional-scale response to external forcings (37).

### Grounding-Zone Position During the Holocene.

Cores KC15 and KC19 are unique because they are the sedimentary records closest to the modern Thwaites Glacier grounding zone that have been radiocarbon dated. Previous work on marine sediment cores had established that the grounding zone of Thwaites Glacier had retreated to within ca. 100 km of its present-day position by approximately 10.3 cal. ka BP (19). This is also in agreement with cosmogenic exposure dating at Mt. Murphy to the west, which shows that rapid ice sheet thinning following the last glacial maximum (LGM) took place predominantly in the early Holocene (47). Radiocarbon ages on calcareous microfossils from glaciomarine sediments near the bases of cores KC15 and KC19 are consistent with this regional timeline but, importantly, reveal that grounded ice had retreated to within ca. 45 km of the modern grounding zone already prior to ca. 9.4 cal. ka BP (Fig. 4 and *SI Appendix*, Figs. S3 and S4 and Table S2).

The grain size distributions, magnetic susceptibility data, and lithological successions (revealed especially by the CT scans) of cores KC15 and KC19 are nearly identical, even though the sites are 1 km apart and in different water depths (545 m and 704 m, respectively). These two core sites experienced similar oceanographic and depositional conditions for an extended period of time, i.e., over about 9 kyrs (NB: modern deposition at site KC15 is documented by the ^210^Pb profile for core MC16, see *SI Appendix*, Fig. S6). The transition from Facies 2 to Facies 1, which occurs at about 1 m below the seafloor in both cores KC15 and KC19 (Fig. 4), can be attributed to two possible competing scenarios, depending on the interpretation of Facies 2: If Facies 2 represents a sub-ice shelf environment, this transition reflects the retreat of Thwaites Glacier’s ice shelf (and grounding zone), which has been ongoing during the last several decades or longer (6, 48), after the grounding zone and calving-line positions had remained largely unchanged over the preceding ca. 9 kyrs. Alternatively, this transition may indicate that the ice-shelf front and possibly the grounding zone were located farther south than today during the deposition of Facies 2 and readvanced at some time in the Holocene, which led to the deposition of Facies 1. A new ice volume history for the eastern Amundsen Sea drainage sector reconstructed from relative sea-level changes in Pine Island Bay concluded that there has been no major ice loss from the mid-Holocene until recently, and that significant grounded-ice retreat beyond the present configuration during this time was unlikely (21). Similarly, proxies for CDW advection towards the ice margin in Pine Island Bay, which is considered as the main external driver of current glacial retreat in the region (49), indicate intensified CDW advection between at least 10.4 and 7.5 cal. ka BP and then again since the mid-twentieth century but with only reduced CDW forcing in between (50, 51). This finding also suggests that significant ice loss during the middle-late Holocene is unlikely. Moreover, core sites KC15 and KC19 were located within the shear zone between the Thwaites Glacier Tongue and the Eastern Ice Shelf before recent retreat of the ice-shelf front (Fig. 1) (52). The history of this shear zone throughout the Holocene is not established, but observations in recent years showed that the calving front here may have shifted further south (52, 53), which may also have happened during the mid-late Holocene and resulted in seasonally open waters above sites KC15 and KC19. In the lower half of these two cores, clay mineral data indicate that Facies 2 has a component of Pine Island Glacier sourced sediments. Although uncertainty remains about the exact depositional setting for Facies 2 in cores KC15 and KC19, both records document that i) grounded ice had retreated from Thwaites Glacier’s present ice margin already by 9.4 cal. ka BP, and ii) the positions of the glacier’s grounding zone and ice shelf front underwent only minor oscillations, if any, over the following ca. 9 kyrs until recent retreat started. These older sediments highlight that the accelerated, major ice loss observed today is uncommon or even unprecedented within a Holocene context.

### Twentieth Century Ice-Shelf Thinning and Grounding-Zone Retreat.

These new core data reveal when Thwaites Glacier’s recent phase of ice-shelf thinning and retreat began. ^210^Pb chronology constrains the timing of facies shifts documenting ice-shelf unpinning from the seafloor and/or grounding-zone retreat. A downcore decrease in excess ^210^Pb activity is observed in most cores, except KC15 and KC19 (*SI Appendix*, Figs. S3 and S4). Major facies shifts that occurred within the past century are archived in cores KC04, JGC11/MC12, KC13, MC16 (same site as KC15) and KC23 along the entire Thwaites Glacier front (Fig. 1). Deeper sites closer to the modern grounding zone, JGC11/MC12 and KC23, record grounding-zone retreat. The shallowest sites KC04, KC13, and JGC17 record ice-shelf thinning and unpinning from seafloor highs, H2 and H1.

Cores JGC11/MC12 and KC23 collected from deeper sites on the western and eastern margins of the Thwaites Glacier ice-shelf system, record prominent shifts from coarse-grained Facies 4 to fine-grained Facies 1. The cessation of mass flow deposition (Facies 4) may indicate retreat of the grounding zone followed by meltwater plume deposition (Facies 1). Other cores from the eastern Amundsen Sea Embayment, which collected sedimentary sequences deposited from the last glacial period to the Holocene, show a similar facies succession that has been interpreted as evidence for grounding-zone retreat (18, 22). According to the ^210^Pb chronology, the deposition of Facies 1 sediments in MC12 (same site as JGC11) started around 1958 C.E. ± 11 y (Fig. 4 and *SI Appendix*, Fig. S6). About 4 km southeast of cores JGC11 and MC12, a high-resolution survey of the seafloor reveals geomorphic evidence for past grounded ice (54). Glacial retreat features found in this survey indicate rapid grounded-ice retreat (ca. 2 km/y) sometime within the past two centuries. The facies succession is more complex at site KC23. There are sharp boundaries between the succession of Facies 4, 3, and 1 (*SI Appendix*, Fig. S5) that we interpret as recording distal glaciomarine deposition progressively replacing deposition in a setting proximal to the grounding zone. Therefore, the bed of Facies 3 may represent an intermediate sub-ice shelf setting at this specific location during retreat that has no equivalent in other cores due to more rapid grounded-ice retreat. The presence of excess ^210^Pb activity in the uppermost sediments from KC23 indicates that the Facies 1 and 3 beds accumulated sometime in the past 150 y (*SI Appendix*, Fig. S5).

**Fig. 6.**
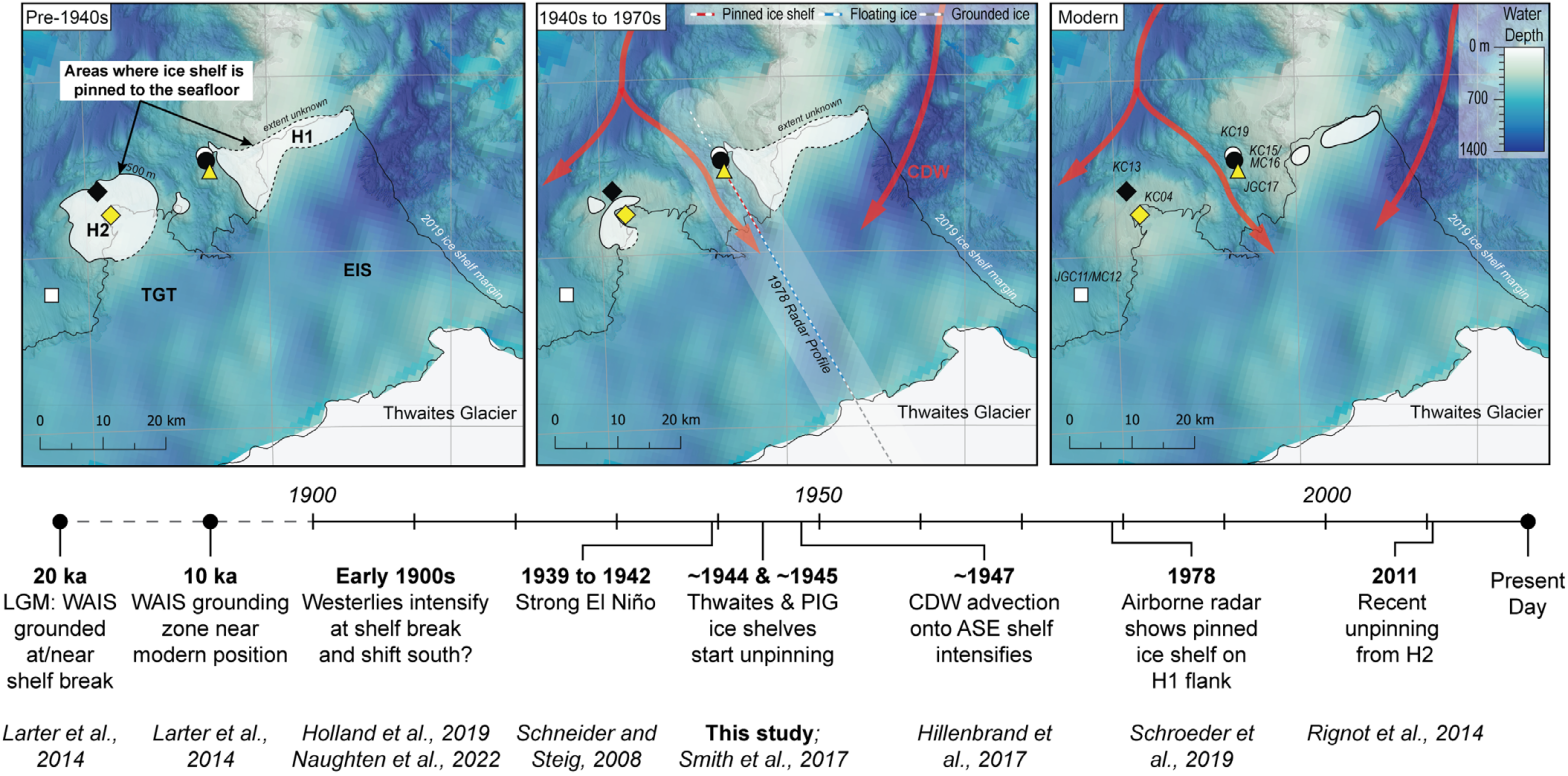
Unpinning of the Thwaites Glacier Tongue (TGT) and Eastern Ice Shelf (EIS) from seafloor highs, previously named H1 and H2, during the twentieth century (panels above) in the context of the long-term climate and glaciological history of the Amundsen Sea drainage sector of the WAIS (timeline below; ASE: Amundsen Sea Embayment; PIG: Pine Island Glacier) (10, 13, 16, 37, 50, 56–58). White polygons in each panel illustrate areas where Thwaites Glacier is grounded and where the ice shelf is pinned to seafloor highs. Note that the ice shelf is floating between the grounding zone and pinning points. The white polygon beneath the ‘Thwaites Glacier’ label indicates approximate modern grounded ice extent in the south (bottom of the maps). The earliest satellite-based grounding-zone locations are from the 1990s (33). The radar profile implies that the grounding zone (gray and white line in the *Middle* panel) was advanced only ~5 km north in 1978 (13). Reconstructions are based on seafloor bathymetry, core chronology, airborne radar surveys, and satellite data (10, 13, 15, 37, 50). Water depth and core site names are shown in the *Right* panel. The intensification of westerlies in the early 1900s is noted with a question mark because some climate models do not clearly support this trend over the past century (59).

Remote sensing datasets indicate that the Thwaites Glacier Tongue had small regions of contact with the H2 summit in the 1990s and had lost all contact after 2011 C.E. and experienced significant structural weakening since the 1990s (Fig. 1) (10, 33, 55). Cores KC04 and KC13 show that changes in ice-shelf configuration started as early as the mid-twentieth century. The cores collected from seafloor highs H1 and H2 reflect a change from sub-ice shelf sediment accumulation in the vicinity of a pinned ice shelf (Facies 5 intercalated with Facies 1 and 3, and Facies 4 intercalated with Facies 5, respectively) into purely glaciomarine deposition (Facies 1) (Figs. 2 and 3 and *SI Appendix*, Fig. S2). Core KC04 was recovered from a narrow, protected basin near the H2 summit (Fig. 5*A*). The excess ^210^Pb profiles for cores KC04 and KC13 are nearly identical, suggesting that the same depositional conditions were shared across both sites for the past 70 y. Both cores show an abrupt shift from Facies 5 to Facies 1 at about 30 cm core depth, which represents ice-shelf cavity expansion and nearby loss of pinning. The ^210^Pb age model for core KC04 indicates the Thwaites Glacier Tongue was pinned closer to this site prior to 1944 C.E. ± 12 y and has decoupled from the seafloor since then (Fig. 6). The sediment record at the KC04 core site may also capture twentieth century ice-shelf changes for Pine Island Glacier. An IRD layer in KC04 possibly records a large calving event from Pine Island Glacier between 1966 and 1973 (*SI Appendix*, Text) (39). The lower part of KC04 includes fine-grained layers within Facies 5 and beds of Facies 1 and 3, which imply that brief episodes of ice-shelf decoupling from the seabed had occurred prior to the 1940s.

Only a portion of the Eastern Ice Shelf is pinned on the H1 high today (14). Satellite radar interferometry and aerogravity studies have documented the ice shelf gradually losing contact with the seafloor, which has resulted in accelerated ice flow (9, 10, 60). Airborne radar profiles collected in 1978 C.E. and 2009 C.E. show thinning of the Eastern Ice Shelf and unpinning from the south-western flank of H1 over that timespan (13). The transect of cores JGC17, KC15/MC16, and KC19 was collected along the same path as the 1978 radar line (Fig. 1), providing valuable observational data to corroborate the shallow sedimentary record below the ice shelf. Site JGC17 is located on a relatively flat-topped summit. This feature is not unique as similar features had been mapped immediately seaward along the Thwaites Glacier ice-shelf system by Hogan et al. (15), who suggested that the pinned ice shelf had sculpted this morphology on seafloor highs in the past when the ice shelf was thicker and more advanced (Fig. 5*B*). The ice-shelf unpinning, recorded in the shift from Facies 4 to 1, occurred sometime within the twentieth century or possibly earlier. The ^210^Pb data in core JGC17 cannot provide the exact timing of the ice-shelf unpinning from that location on H1 (*Materials and Methods*); however, the presence of excess ^210^Pb activity above background levels indicates that the fine-grained Facies 1 at the top of this core accumulated sometime within the past century.

### Regional Glacial History and Potential Drivers of Change.

Regional trends in grounding-zone retreat in the Amundsen Sea have been observed since the 1970s (5). However, with sparse data coverage prior to satellite-based measurements, it has been difficult to decipher the spatial and temporal trends in glacier retreat or establish the driver, i.e., internal vs. external variability. Our results indicate that Thwaites Glacier started to unpin and retreat in the mid-twentieth century, and together with previously published records from neighboring Pine Island Glacier, provide greater certainty about the timing of glacier retreat in this climatologically sensitive region. Analyses of sub-ice-shelf cores from Pine Island Glacier ice shelf revealed grounding-zone retreat and cavity expansion started around 1945 C.E. ± 12 y, with final unpinning of the ice shelf from a prominent seafloor ridge taking place at ~1970 C.E. ± 4 y (37). The synchronous retreat of Thwaites and Pine Island glaciers in the mid-twentieth century implies that both ice streams were responding to the same driver(s). In this context, prolonged El Niño conditions between 1939 and 1942 C.E. have previously been suggested as the most likely driver of twentieth century retreat of Pine Island Glacier (37, 61), with modelling work showing that El Niño promotes enhanced CDW upwelling onto the continental shelf through increased wind stress at the shelf break (61–63). Indeed, proxy data (stable carbon isotope measurements on planktic foraminifera) from a core site within the eastern Pine Island Trough indicate enhanced CDW advection around 1947 C.E. ± 9 y (50), providing a link between climate variability in the tropical Pacific, increased CDW on the continental shelf, and the onset of glacier retreat in the Amundsen Sea in the mid-twentieth century (56). Climate-modeling results have shown that Amundsen Sea glacier retreat reflects a long-term strengthening in local westerly winds due to anthropogenic radiative forcing (57, 58). However, data-constrained reconstructions indicate that the local shelf-break winds have an easterly, rather than westerly, trend over the last century (59, 64). Our results thus support the idea that glacier retreat in the Amundsen Sea was initiated by natural climate variability in the 1940s (61). That ice streams such as Thwaites Glacier and Pine Island Glacier have continued to retreat since then indicates that they were unable to recover after the exceptionally large El Niño event of the 1940s (64). This may reflect the increasing dominance of anthropogenic forcing since that time (64) but implies that this involved large-scale, in additional to local, atmospheric and ocean circulation changes.

## Materials and Methods

A full description of methods, including data collection in the field, is included in the *SI Appendix*, Text File.

Computed tomography (CT) scans of sediment cores were collected at the Oregon State University College of Veterinary Medicine using a Toshiba Aquillion 64 Slice. The data were processed using the MATLAB package SedCT (65). To characterize the sedimentary facies downcore, discrete sediment samples were analyzed for grain size distribution and grain shape with a CILAS 1190 laser particle size analyzer, which measures grains between 0.4 and 2000 μm. When larger clasts were present, the abundance was approximated through manual pebble counting on the CT scans across 5-cm depth intervals. The CILAS is connected to a microscope to take pictures of sand-sized grains. From these photos, individual quartz grains were extracted as binary images using ExpertShape software. The binary grain images were processed with the MATLAB package MORPHEOLV to calculate the roughness coefficient for each grain in the sample interval (35).

The clay fraction <2 µm was separated from the bulk sediment by settling and then used to determine the relative contents of the clay minerals smectite, illite, chlorite, and kaolinite. Samples were analyzed with an automated powder diffractometer system Rigaku MiniFlex with CoKα radiation (30 kV, 15 mA) at the Institute for Geophysics and Geology, University of Leipzig, Germany.

^210^Pb and ^14^C measurements were employed to date the sediments deposited during the last ca. 150 to 200 y and the Holocene, respectively. The ^210^Pb dating was conducted at the Department of Earth and Atmospheric Sciences, University of Houston. A Canberra broad-energy germanium gamma-ray spectrometer was used. For dating older units, bulk sediment samples were taken systematically down-core, wet sieved to isolate the >63 µm fraction, and investigated under a microscope for the presence of calcareous foraminifera. Calcareous foraminifera were sent to the Laboratory of Ion Beam Physics, ETH Zürich, Switzerland, for MICADAS radiocarbon dating. See *SI Appendix*, Table S2 for details on radiocarbon calibration.

## Supplementary Material

Appendix 01 (PDF)

## Data Availability

Down-core sedimentological data have been deposited in PANGAEA Database for Earth and Environmental Science. All data will be available online in the PANGAEA Database for Earth and Environmental Science (https://doi.org/10.1594/PANGAEA.964530).
