# Supplementary material for: Synchronous retreat of Thwaites and Pine Island glaciers in response to external forcings in the presatellite era
Source: Proc Natl Acad Sci U S A. 2024 Feb 26;121(11):e2211711120. doi: 10.1073/pnas.2211711120 (PMC10945778; doi:10.1073/pnas.2211711120)
Supplement: Supplementary file 1 — Appendix 01 (PDF) [file pnas.2211711120.sapp.pdf]

PNAS

Supplementary Information for

Synchronous retreat of Thwaites and Pine Island glaciers in response to external forcings in the pre-satellite era

Rachel W. Clark[a], Julia S. Wellner[a], Claus-Dieter Hillenbrand[b], Rebecca L. Totten[c], James A. Smith[b], Lauren E. Miller[d], Robert D. Larter[b], Kelly A. Hogan[b], Alastair G.C. Graham[e], Frank O. Nitsche[f], Asmara A. Lehrmann[c], Allison P. Lepp[d], James D. Kirkham[b,g], Victoria T. Fitzgerald[c], Georgina Garcia-Barrera[a], Werner Ehrmann[h], Lukas Wacker[i]

[a]Department of Earth and Atmospheric Sciences, University of Houston, Houston, TX 77004

[b]British Antarctic Survey, Cambridge, CB3 0ET, UK

[c]Department of Geological Sciences, University of Alabama, Tuscaloosa, AL 35401

[d]Department of Environmental Sciences, University of Virginia, Charlottesville, VA 22903

[e]College of Marine Science, University of South Florida, St. Petersburg, FL 33701

[f]Lamont-Doherty Earth Observatory of Columbia University, Palisades, NY 10964

[g]Scott Polar Research Institute, University of Cambridge, Lensfield Road, Cambridge, CB2 1ER, UK

[h]Institute for Geophysics & Geology, University of Leipzig, Leipzig 04103, Germany

[i]Ion Beam Physics, ETH Zürich, Zürich, Switzerland

Corresponding authors: Rachel W. Clark & Julia S. Wellner

Email: rwclark2@central.uh.edu, jswellne@central.uh.edu

This PDF file includes:

Supplementary Information Text and References
Figures S1 to S6
Tables S1 to S3

Supplementary Information Text

Materials and Methods

Data Collection

During the NBP19-02 cruise, swath bathymetry and acoustic sub-bottom profiler surveys were conducted throughout the cruise, to map glacial geomorphic features and to identify coring sites. Core sites were chosen where stratified subseafloor sediments were apparent in the sub-bottom profiles, and often particular geomorphic features were targeted, such as channels, sediment wedges, and sediment infill in basins located atop basement highs.

Aboard the vessel, KCs were photographed and sediment lithology, sedimentary structures, and Munsell colors were recorded. Smear slides, discrete sediment samples, and archive sections were collected from the KCs. MCs were cut continuously downcore into 1 cm sample slices, while JGCs were cut into 1.5 m long sections and sent to the Marine and Geology Repository at Oregon State University (OSU-MGR), U.S.A., for additional physical properties analyses and discrete sampling. Unless stated otherwise, all discrete sediment samples used in this study were analyzed post-cruise at the Department of Earth and Atmospheric Sciences, University of Houston, U.S.A.. All sediment cores and discrete samples were stored between +2 °C and +4 °C after collection.

Analysis of Physical Properties

Shear strength was measured with a hand-held shear vane either onboard (KCs) or post-cruise immediately after splitting (JGCs). These analyses were conducted at 10 cm depth resolution, except in very soupy sediment intervals near core tops. Additional physical properties were measured on the JGC core sections and KC archives trays at the OSU-MGR. Prior to splitting and sampling the JGC sections were analyzed at 1 cm depth resolution with a GEOTEK multi-sensor core logger (MSCL-S) for p-wave velocity, wet bulk density, and whole core magnetic susceptibility. Both JGC archive halves and KC archive trays were digitally photo-scanned with a line-scan camera and analyzed for high-resolution magnetic susceptibility at 1 cm depth resolution using a GEOTEK XZ split core track. In some core sections, unreliable magnetic susceptibility data caused by an uneven core surface or voids were removed.

Computed tomography (CT) scans were also collected from selected cores at the Oregon State University College of Veterinary Medicine using a Toshiba Aquillion 64 Slice. The data were processed using the MATLAB package SedCT (1). The SedCT package provides a dimensionless value representative of the sediment that summarizes the CT scan downcore. This dimensionless value, known as the CT number or Hounsfield Unit, is a density proxy and calculated using the attenuation coefficients (μ) for water and the sample data (Formula 1) (2). The CT number for water is 0 and that for air is -1000 (1). Overall, the sediment cores exhibit values between 800 and 2100, where an increasing CT number corresponds to increasing particle density/lower water content. Measurements were collected every 0.5 mm downcore, so the CT number plots are shown using a ten-point moving average to optimize visualization of downcore trends. The CT scan images are presented in false color and grayscale to highlight as much internal detail as possible.

Formula 1: Hounsfield Unit $= (\mu_{sample} - \mu_{water}) / \mu_{water} \times 1000$

Grain size and shape measurements

To characterize the sedimentary facies downcore, discrete sediment samples were analyzed for grain size distribution and grain shape with a CILAS 1190 laser particle size analyzer , which measures grains between 0.4 and 2000 μm. These data are presented using the

Wentworth grain size classification scheme, where the maximum diameter for sand is 2000 μm, 63 μm for coarse silt, 31 μm for medium silt, 16 μm for fine silt, 8 μm for very fine silt, and 3.9 μm for clay (3). When clasts larger than 2000 μm were present, their abundance was approximated through pebble counting on the CT scans across 5 cm intervals. The CILAS is also connected to a microscope to take pictures of sand-sized grains. From these photos, individual quartz grains were extracted as binary images using ExpertShape software. The binary grain images were processed with the MATLAB package MORPHEOLV to calculate the roughness coefficient for each grain in the sample interval (4). The mean particle roughness values range between 0.005 to 0.008. Higher roughness values correspond to populations with more angular grains, while lower roughness values correspond to populations with rounder grains. This proxy can be used to interpret past transport processes (4–6).

Clay mineral analyses

The clay fraction <2 μm was separated from the bulk sediment by settling and then used to determine the relative contents of the clay minerals smectite, illite, chlorite and kaolinite. The measurements were conducted with an automated powder diffractometer system Rigaku MiniFlex with CoKα radiation (30 kV, 15 mA) at the Institute for Geophysics and Geology, University of Leipzig, Germany. The clay mineral identification and quantification followed standard X-ray diffraction methods (25). Clay mineral provenance data is presented in the supplement (**Table S3).**

^{210}Pb and ^{14}C dating

^{210}Pb and ^{14}C measurements were employed to date the sediments deposited during the last ca. 150-200 years and the Holocene, respectively. The ^{210}Pb dating was conducted at the Department of Earth and Atmospheric Sciences, University of Houston. The shorter lived ^{210}Pb isotope has a half-life of 22.3 yrs (7). A Canberra broad-energy germanium gamma-ray spectrometer was used to detect low concentrations of ^{210}Pb in discrete sediment samples. Dried sediment samples were homogenized and scooped into polystyrene petri dishes. The petri dishes were stored for at least three weeks prior to measurement so the isotopes could reach radioactive equilibrium (7). Each sample was measured for 20 hours, and the spectral data peaks were analyzed using Genie 2000 spectroscopy software to identify radioactive isotopes. Gamma radiation from ^{210}Pb generates an energy peak at 46.54 keV (8). Background activity, which usually remains constant within a core, is indirectly calculated from the average of ^{214}Pb and ^{214}Bi activity in each sample which approximates the amount of the parent isotope ^{226}Ra in the sediment (9). The sediment in the petri dish naturally absorbs part of the gamma radiation so that the detector cannot measure the total ^{210}Pb activity in the sample. Thus, a ^{210}Pb point-source correction was used to calculate the total activity in the sample (10). It should be noted that a spike in ^{137}Cs activity from nuclear testing in the mid-20th century was not detected in any of the samples for this study. This isotope can provide an independent verification of the ^{210}Pb age models. However, it is common for ^{137}Cs activity to be below the limit of instrument detection in sediments collected at this far south. Bioturbation can modify downcore, excess ^{210}Pb activity, which would could result in younger sediments being mixed into deeper layers (11). The CT scans reveal undisturbed, laminated sediments without any bioturbation in most core tops where excess ^{210}Pb is detected (e.g., KC04).

^{210}Pb isotope activity measurements were normalized to the weight of the sediment so that the isotope concentrations are presented in Becquerels per gram (Bq/g), where one Becquerel equals one disintegration per second. The excess activity in the sample, often called “unsupported activity” in other studies, is isolated when subtracting the background activity from the total ^{210}Pb activity measured in the sample (7, 12). In general, excess activity is highest at the sediment/water interface and decreases downcore until reaching background levels. A regression line is fit to the data to estimate the sediment accumulation rate in each core (11). When possible, the timing of sediment accumulation within a core is determined using a model. For cores with the

excess activity systematically decreasing downcore, the Constant Initial Concentration (C.I.C.) model is employed to infer the age of deposition (7, 12). This model assumes that the amount of ^{210}Pb input has remained constant throughout the time interval of deposition. Age uncertainty, calculated from the standard deviation of ages, ranges between 0 (assumed at the surface) to ~10 years and typically increases downcore. Additional uncertainty which can be difficult to quantify may stem from the individual data point error bars, the physical spacing down core between dated samples, and the sedimentation rate or actual age separation between samples. Some cores did not show systematic ^{210}Pb decay in the uppermost sediment column, possibly due to very slow sediment accumulation rates or seafloor surface sediment loss during core recovery. For these cases, the data are still presented, even though they could not be used to infer the age of deposition or the sediment accumulation rate.

Measurement of ^{210}Pb activity was performed in the uppermost sediments at all core sites. The ^{210}Pb data presented here were primarily collected from Facies 1, but a few measurements were taken from Facies 3, 4 and 5, when they were present near the seafloor surface. Excess ^{210}Pb was detected in most core top sediments, except KC15 and KC19 (**Fig. S3, S4**). Excess ^{210}Pb activity was measured downcore until the excess activity reached background values, i.e., as far as 30 cm core depth at site KC04. KC04 and KC13 show nearly identical downcore ^{210}Pb profiles (**Figs. 2, S2**). For JGC11, JGC17, and KC23, the presence of excess ^{210}Pb activity above background levels indicates the uppermost sediments accumulated sometime in the past 150 years based on the short ^{210}Pb half-life and instrument detection limits; however, the unusual excess activity decay curves prevent age model calculation at this time. In core KC23, the unusual decay curve is likely related to a major lithological change in the upper 10 cm at this site (i.e., Facies 3 underlying Facies 1). The ^{210}Pb data for core JGC11 and JGC17 shows excess activity in the uppermost Facies 1 sediments (**Fig. 3**), but no accumulation rate or age model could be calculated for this site because the uppermost sediments were slightly disturbed during the core recovery process.

For dating older units, bulk sediment samples were taken systematically downcore, wet sieved to isolate the >63 µm fraction, and investigated under a microscope for the presence of calcareous foraminifera, which provide the most reliable ^{14}C ages in Antarctic shelf sediments (13). Picked calcareous benthic and planktic foraminifera (900 - 1200 µg) were sent to the Laboratory of Ion Beam Physics, ETH Zürich, Switzerland, for MICADAS radiocarbon dating with a gas ion source (14). Calcareous microfossils are rare in Antarctic shelf sediments, so only a few ^{14}C ages are presented in this study. Radiocarbon ages were calibrated for Marine20 for low depletion scenario with CALIB 8.2. using a ΔR value of 843 ± 40 years and using the Marine20 calibration curve (15–17). This ΔR is averaged from the two nearest Antarctic continental shelf sites in the CALIB Marine Reservoir Correction Database, sites 521 (873 ± 39) and 522 (813 ± 40) in the Antarctic Peninsula. Calcareous fossils are rare in the sediments; therefore, we present limited radiocarbon ages. Our initial goal was to date the base of the cores to determine the maximum timespan recorded in each core site. Radiocarbon dating of benthic foraminifera from KC15 and KC19 produced early Holocene ages near the base of these cores (**Fig. 4; Table S2**). Benthic foraminifera in KC04 produced young ages from about three centuries before present. See **Table S2** for details on radiocarbon calibration.

Detailed Facies Descriptions.

Facies 1 is a laminated, very soft clayey silt with high water content (>30 wt. %) that decreases downcore and has low shear strength (<2 kPa). In some acoustic sub-bottom profiles, this facies corresponds to a thin semi-transparent drape, for example at site KC19 (**Fig. 5**). Facies 1 can be up to 50 cm thick, is pervasive across the study area, and is present at the seabed surface in all cores. We applied ^{210}Pb dating the uppermost sediments, which are primarily Facies 1 (**Fig. 4**). Facies 1 has a mean grain size of 10 µm with discrete measurements

showing subtle variations in silt content. CT scans reveal thin, subtle laminations, which had been visually undetectable. The CT number ranges from 700 to 950 Hounsfield Units (HU), reflecting low density, water-rich sediment. Matrix-supported pebbles and/or sand grains are present in the upper 10 cm of this facies at sites KC04, JGC11, and KC13 (**Figs. 2, S1, S2**). Sandy laminae occur in Facies 1 in cores KC15, JGC17, and KC19 (**Figs. 3, S3, S4**). The average coefficient of particle roughness is typically lower in Facies 1 (<0.007) and values occupy a slightly narrower range compared to other facies. Occasionally, whole or fragmented diatom frustules are present, mainly in the upper 10 cm. Calcareous and agglutinated benthic foraminifera tests may also be found in Facies 1. In some cores, the Facies 1 sediments were so soft and soupy that no shear strength measurement could be collected. In general, physical properties vary only slightly for Facies 1, except in the presence of increased sand or gravel content. Magnetic susceptibility is relatively low and shows little variability. Wet-bulk density measured on cores JGC11 and JGC17 is relatively low, varying ~1.5 g/cm^3 (**Figs. 3, and S1**). At the base of KC13, there is a section of sediment that is highly faulted but is most similar in characteristic to Facies 1 and 5 (**Table 1; Fig. 4**). Faulting is the result of either natural processes (e.g., gravitational instability) or coring disturbance. Because of the ambiguity in interpreting this section, it is excluded from the detailed interpretations in the main text (**See Fig. S2 for section**).

Facies 2 is only present in cores KC15 and KC19, where it is more than 2 m thick. The sedimentological traits of Facies 2 appear similar to those of Facies 1, but the mean grain size of Facies 2 is slightly finer (ca. 7 µm), and it rarely bears sand or pebbles-sized grains. Visually and in CT scans, this facies typically shows centimeter-scale, elongate or round mottles, likely associated with bioturbation. Occasional faint or discontinuous, slightly coarser grained laminations are present (**Figs. S3, S4**). The particle roughness coefficient is highly variable in Facies 2 (0.0056 to 0.0077), indicating that grain angularity varies throughout this unit. Shear strength values are low and remain close to 2 kPa, while water content is typically around 40 wt. %. Magnetic susceptibility and CT number values are lower than Facies 1 and increase where slightly coarser laminae occur. Benthic agglutinated foraminifera are common within this facies. Near the base of core KC15 and KC19, a few samples contained calcareous foraminifera that were suitable for radiocarbon dating (**Table S2**).

Facies 3 is relatively thin (≤ 10 cm thick). It is observed in cores KC04, KC15, KC19, and KC23, which were recovered at different distances from the modern ice-shelf front and from different water depths. This laminated facies is a sandy silt with a mean grain size varying from 17 to 42 µm. Sand content mainly ranges between 10 and 39 %, with one sample in KC23 displaying a maximum sand content value of 50 % (**Fig. S5**). The range of particle roughness values is relatively narrow and values are high, similar to Facies 4 and 5(>0.0069) (**Table 1**). Thin interbeds, resembling Facies 3, occur within Facies 1 and 5 (**Figs. 2, S3-S5**). The CT numbers are typically high, exceeding 1000 HU. In comparison to all other facies, Facies 3 has low to medium magnetic susceptibility values. Water content ranges between 26 and 37 wt. %.

Facies 4, which is observed in JGC11, JGC17, and KC23, contains abundant pebbles unlike Facies 1-3 (**Figs. 3, S1, S5**). This facies is a predominantly massive diamicton, barren of any microfossils. Mean grain sizes within the matrix material (i.e., the <2 mm fraction) range between 11 and 45 µm. Grain roughness ranges in this facies, but most values are consistently higher (> 0.007) when compared to Facies 1 (e.g. JGC11 in Fig S1). In KC23, the particle roughness value are consistently high and uniform in the lower half of the core (**Fig. S5**). Other cores show high variability of particle roughness in Facies 4, such as JGC11 (**Fig S1**). Shear strength is usually low (~2 kPa) but reaches up to 24.5 kPa at the base of JGC11. Water content in Facies 4 is as low as 10 wt. %, but wet-bulk density is higher and ranges from 1.75 to 2.25 g/cm^3. Magnetic susceptibility is highly variable (50 to 800 x10^{-5} SI units). Facies 4 is characterized by maximum CT numbers throughout, reflecting very high density, especially where large cobbles are present. Discrete zones with a variable clast-to-matrix ratio are identified (e.g.,

from 60 to 100 cm depth in core JGC11, **Figs. 4, S1**), but there does not seem to be a dominant clast orientation within this facies. Both JGC11 and KC23 include single, thick beds of Facies 4, while JGC17 includes three distinct beds alternating with two Facies 5 beds. Facies 4 in JGC11 displays fining upward in the CT scan image, which is also evident from a downcore increase in both the coarse clast abundance and the sand content as well as average grain size within the matrix fraction (**Fig. S1**). A coinciding downcore increase in the CT number of JGC11 is observed, too, but this could simply reflect sediment compaction. Facies 4 sediments of core KC23 also appear to be characterized by a fining upward trend, but it is much less prominent. Here, Facies 4 can be divided into an upper and lower diamicton, with the matrix of the lower diamicton below ca. 0.9 m core depth having a slightly higher sand content, resulting in coarser mean grain size (**Fig. S5**).

Facies 5 exhibits the most variability amongst the five facies and is classified as stratified diamicton with thin, alternating interbeds of fine-grained sediments resembling Facies 1 and 3. Facies 5 is identified in cores KC04, KC13, and JGC17, which are all located on seafloor highs H1 and H2 (**Figs. 2, 3, S2**). The grain size distribution is variable within this facies, with the mean grain size within the <2 mm fraction ranging from 10 to 21 µm. Shear strength is low in both the gravel-rich beds and interbeds (≤2 kPa), while water content is slightly higher than in Facies 4. Values for magnetic susceptibility, wet-bulk density, CT number, and water content vary widely in Facies 5, but they generally do not exceed the values measured for Facies 4. CT scans reveal that the long axes of the larger clasts in Facies 5 appear to be horizontal to sub-horizontal. In some instances, either depressions in fine-grained sediments below or draping of mud above larger clasts are observed, e.g., at 179 and 186 cm depth in core KC04, which consists almost entirely of Facies 5 sediments (**Fig. 2**). In KC04, the fine-grained layers, intercalated between the coarse-grained beds, exhibit significant grain-size variability. Where Facies 1 and 3 interbeds are thicker than 10 cm, they are marked in **Figures 2** and **4**, whereas all thinner interbeds were considered to be part of the stratified diamicton. In core JGC17, Facies 5 is identified by thin beds of stratified diamicton, including thin fine-grained layers intercalated with thick, massive Facies 4 beds (**Figs. 3, 4**).

Origin of IRD-rich layer in KC04

A discrete layer of coarse-grained clasts, interpreted as ice-rafted debris (IRD) transported and deposited either by an ice shelf or by individual icebergs, is observed at 7-8 cm below the seafloor in core KC04 (**Fig. 2**). This relative IRD maximum may indicate the transition from sub-ice shelf to open marine conditions during ice-shelf front retreat because icebergs originating from various glacial outlets can be trapped at an ice shelf front by wind, waves or sea ice for a prolonged time (18, 19). The clay mineral provenance of the matrix of this IRD layer is characterized by an illite maximum and a smectite minimum, making it distinct from the clay mineral composition of the underlying, lithologically similar stratified diamictons of Facies 5, which are interpreted as sub-ice shelf sediments (see below). Thus, the clay mineral provenance signature of the IRD layer in KC04 suggests that it was mainly sourced by glacigenic detritus from Pine Island Glacier (20–22). According to the ^{210}Pb chronology, the IRD layer formed sometime between 1961 and 1982 (**Fig. 2**). Based on this chronology, this IRD layer could be recording a large calving event at Pine Island Glacier between 1966 and 1973 (23).

References for Supplement

1. B. T. Reilly, J. S. Stoner, J. Wiest, SedCT: MATLAB™ tools for standardized and quantitative processing of sediment core computed tomography (CT) data collected using a medical CT scanner. *Geochemistry, Geophys. Geosystems* **18**, 3231–3240 (2017).

2. G. N. Hounsfield, Computerized transverse axial scanning (tomography): Part 1. Description of system. *Br. J. Radiol.* **46**, 1016–1022 (1973).

3. C. K. Wentworth, A Scale of Grade and Class Terms for Clastic Sediments. *J. Geol.* **30**, 377–392 (1922).

4. I. Charpentier, A. B. Staszyc, J. S. Wellner, V. Alejandro, Quantifying grain shape with MorpheoLV: A case study using Holocene glacial marine sediments. *EPJ Web Conf.* **140**, 4–7 (2017).

5. D. E. Robinson, J. Menzies, J. S. Wellner, R. W. Clark, Subglacial sediment deformation in the Ross Sea, Antarctica. *Quat. Sci. Adv.* **4**, 100029 (2021).

6. D. N. Livsey, *et al.*, Fourier grain-shape analysis of antarctic marine core: The relative influence of provenance and glacial activity on grain shape. *J. Sediment. Res.* **83**, 80–90 (2013).

7. P. G. Appleby, "Chronostratigraphic techniques in recent sediments" in *Tracking Environmental Change Using Lake Sediments*, W. M. Last, J. P. Smol, Eds. (Springer, 2001), pp. 171–203.

8. G. Gilmore, "Chapter 16: Gamma Spectrometry of Naturally Occurring Radioactive Materials (NORM)" in *Practical Gamma-Ray Spectroscopy*, 2nd Ed., (John Wiley & Sons, 2011), pp. 315–328.

9. W. Tylmann, A. Bonk, T. Goslar, S. Wulf, M. Grosjean, Calibrating 210Pb dating results with varve chronology and independent chronostratigraphic markers: Problems and implications. *Quat. Geochronol.* **32**, 1–10 (2016).

10. G. Haase, D. Tait, A. Wiechen, Determination of full energy peak efficiency for cylindrical volume sources by the use of a point source standard in gamma-spectrometry. *Nucl. Inst. Methods Phys. Res. A* **361**, 240–244 (1995).

11. K. V. Boldt, *et al.*, Modern rates of glacial sediment accumulation along a 15° S-N transect in fjords from the Antarctic Peninsula to southern Chile. *J. Geophys. Res. Earth Surf.* **118**, 2072–2088 (2013).

12. J. A. Sanchez-Cabeza, A. C. Ruiz-Fernandez, 210 Pb sediment radiochronology : An integrated formulation and classification of dating models. *Geochim. Cosmochim. Acta* **82**, 183–200 (2012).

13. C. D. Hillenbrand, *et al.*, Grounding-line retreat of the West Antarctic Ice Sheet from inner Pine Island Bay. *Geology* **41**, 35–38 (2013).

14. L. Wacker, J. Lippold, M. Molnár, H. Schulz, Towards radiocarbon dating of single foraminifera with a gas ion source. *Nucl. Instruments Methods Phys. Res. Sect. B Beam Interact. with Mater. Atoms* **294**, 307–310 (2013).

15. M. Stuiver, P. J. Reimer, CALIB revision 8. *Radiocarbon* **35**, 215–230 (1993).

16. T. J. Heaton, *et al.*, Marine20 - The Marine Radiocarbon Age Calibration Curve (0-55,000 cal BP). *Radiocarbon* **62**, 779–820 (2020).

17. E. L. McClymont, *et al.*, Summer sea-ice variability on the Antarctic margin during the last glacial period reconstructed from snow petrel (Pagodroma nivea) stomach-oil deposits. *Clim. Past* **18**, 381–403 (2022).

18. J. A. Smith, *et al.*, The marine geological imprint of Antarctic ice shelves. *Nat. Commun.* **10**, 5635 (2019).

19. S. Part, E. W. Domack, E. A. Jacobson, J. B. Anderson, Late Pleistocene – Holocene retreat of the West Antarctic Ice-Sheet system in. *New York*, 1517–1536 (1999).

20. J. A. Smith, *et al.*, Sub-ice-shelf sediments record history of twentieth-century retreat of Pine Island Glacier. *Nature* **541**, 77–80 (2017).

21. P. Simões Pereira, *et al.*, The geochemical and mineralogical fingerprint of West Antarctica's weak underbelly: Pine Island and Thwaites glaciers. *Chem. Geol.* **550** (2020).

22. W. Ehrmann, *et al.*, Provenance changes between recent and glacial-time sediments in the Amundsen Sea embayment, West Antarctica: clay mineral assemblage evidence. *Antarct. Sci.* **23**, 471–486 (2011).

23. E. Rignot, Ice-shelf changes in Pine Island Bay, Antarctica, 1947-2000. *J. Glaciol.* **48**, 247–256 (2002).

Supplementary Information Figures

Fig. S1

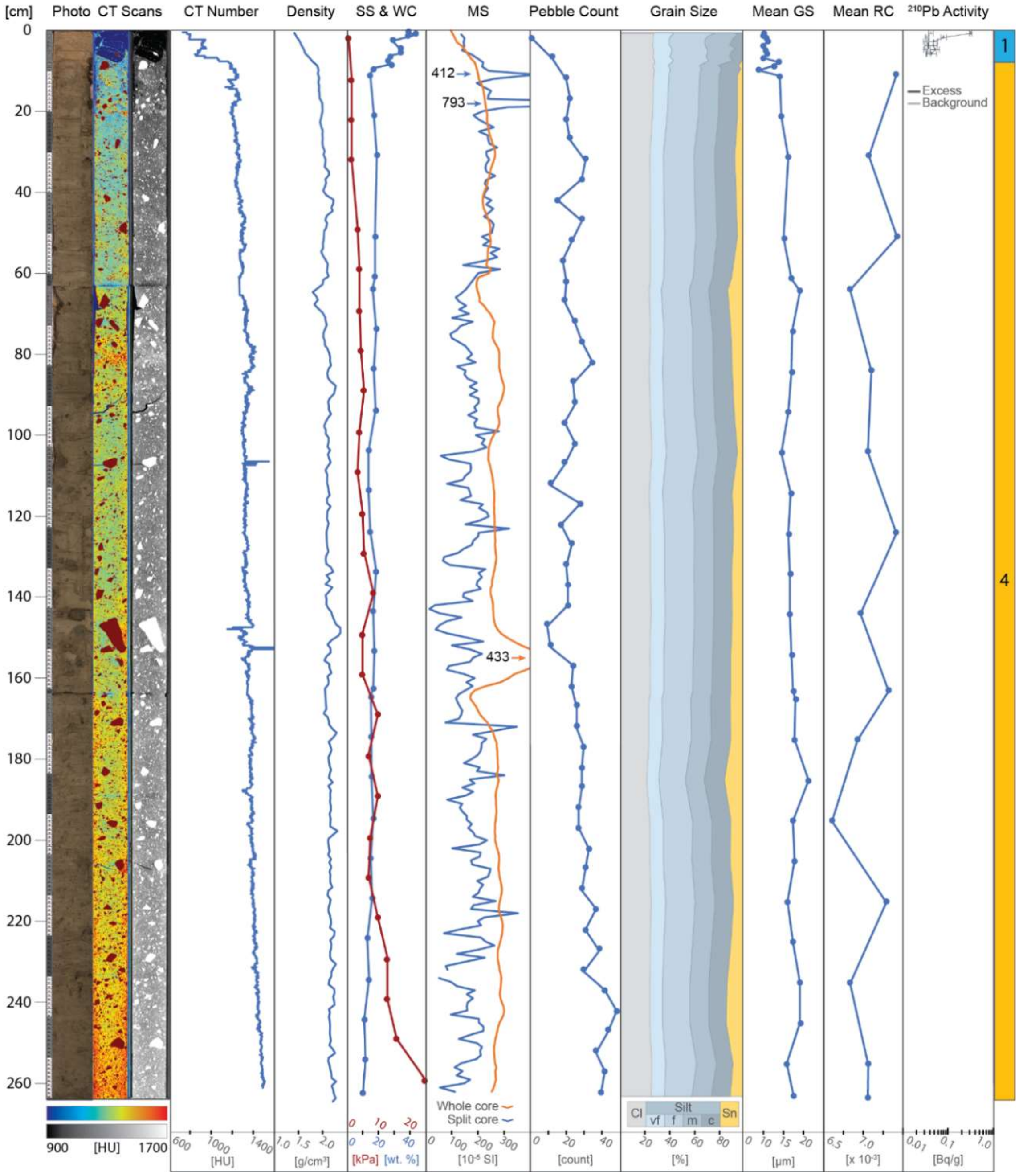

Compilation of proxy data for core JGC11. From left to right: line-scan core image, CT scan images in false color and grayscale (red arrows highlight depressions underneath gravel clasts), CT number, density, shear strength (SS) and water content (WC), point-sensor and whole-core magnetic susceptibility (MS), pebble count, grain size distribution, mean grain size (GS), mean sand grain roughness coefficient (RC), ^{210}Pb activity, and facies assignments, which are defined in Table 1. Error bars are shown for individual background and excess ^{210}Pb activity measurements.

Fig. S2

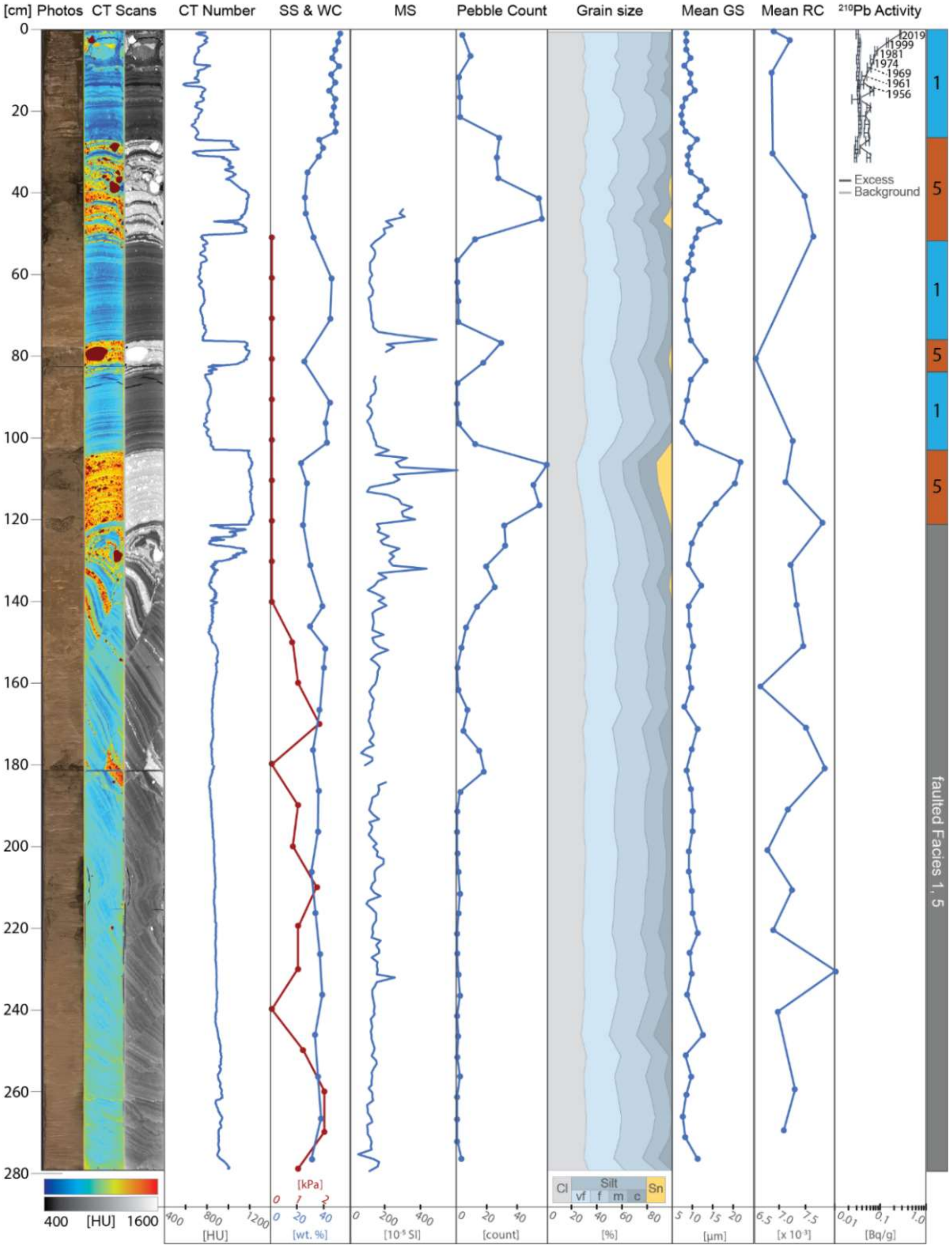

Compilation of proxy data for core KC13. From left to right: line-scan core image, CT scan images in false color and grayscale (red arrows highlight depressions underneath gravel clasts), CT number, shear strength (SS) and water content (WC), point-sensor magnetic susceptibility (MS), pebble count, grain size distribution, mean grain size (GS), mean sand grain roughness coefficient (RC), ^{210}Pb activity, and facies assignments, which are defined in Table 1. Stratigraphy appears faulted between 123 and 279 cm depth (see supplement text). If core surface was uneven, point-sensor MS was not measured. Error bars are shown for individual background and excess ^{210}Pb activity measurements.

Fig. S3

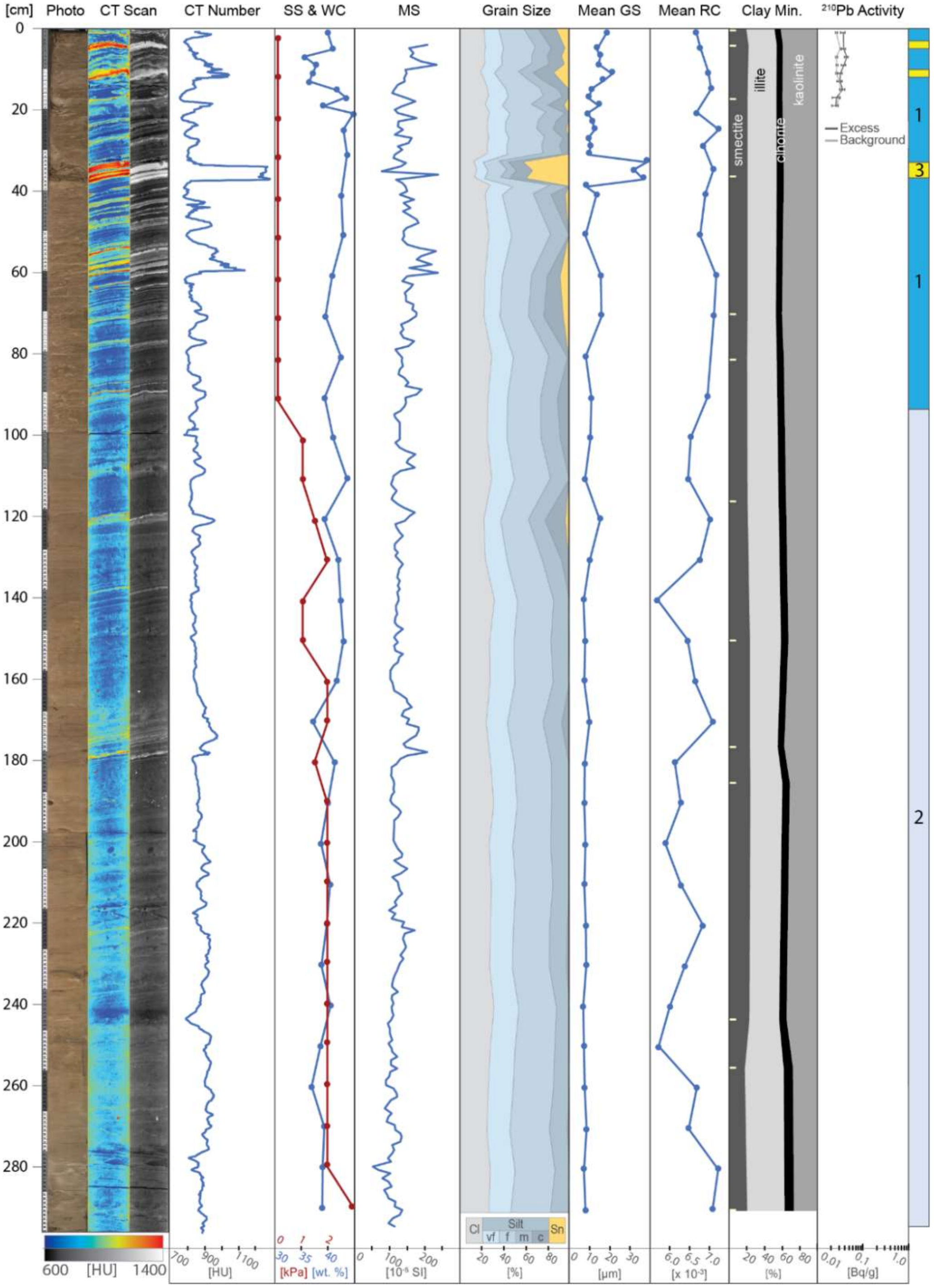

Compilation of proxy data for core KC15. From left to right: line-scan core image, CT scan images in false color and grayscale (red arrows highlight depressions underneath gravel clasts), CT number, shear strength (SS) and water content (WC), point-sensor magnetic susceptibility (MS), grain size distribution, mean grain size (GS), mean sand grain roughness coefficient (RC), clay mineral provenance data (samples indicates with tick marks), ^{210}Pb activity, and facies assignments, which are defined in Table 1. Error bars are shown for individual background and excess ^{210}Pb activity measurements.

Fig. S4

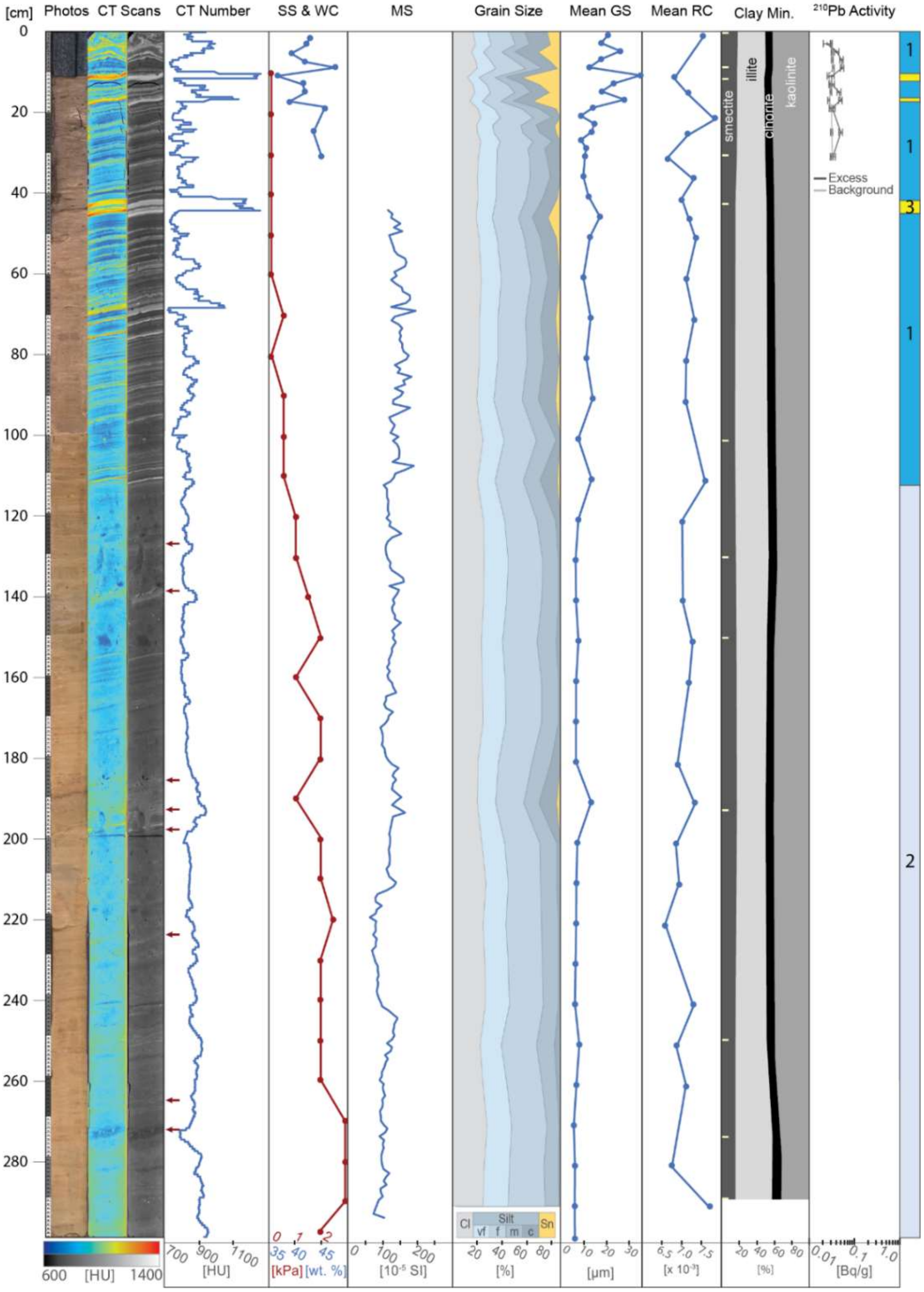

Compilation of proxy data for core KC19. From left to right: line-scan core image, CT scan images in false color and grayscale (red arrows highlight depressions underneath gravel clasts), CT number, shear strength (SS) and water content (WC), point-sensor magnetic susceptibility (MS), grain size distribution, mean grain size (GS), mean sand grain roughness coefficient (RC), clay mineral provenance data (samples indicates with tick marks), ^{210}Pb activity, and facies assignments, which are defined in Table 1. Red arrows indicate examples of partially bioturbated laminations. Point-sensor MS was not measured where core surface was uneven. Error bars are shown for individual background and excess ^{210}Pb activity measurements.

Fig. S5

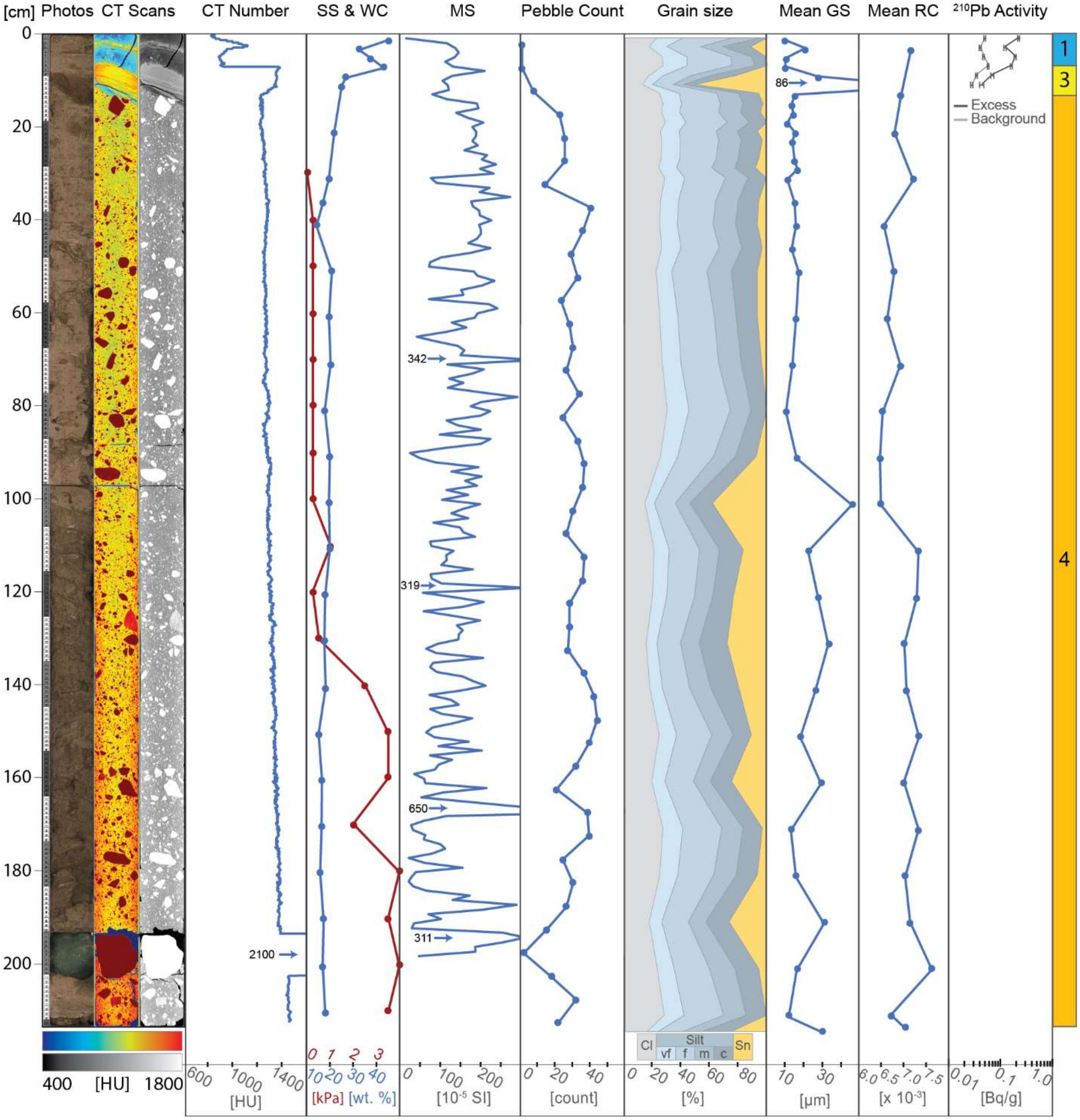

Compilation of proxy data for core KC23. From left to right: line-scan core image, CT scan images in false color and grayscale (red arrows highlight depressions underneath gravel clasts), CT number, shear strength (SS) and water content (WC), point-sensor magnetic susceptibility (MS), pebble count, grain size distribution, mean grain size (GS), mean sand grain roughness coefficient (RC), ^{210}Pb activity, and facies assignments, which are defined in Table 1. Point-sensor MS was not measured where core surface was uneven. Error bars are shown for individual background and excess ^{210}Pb activity measurements.

Fig. S6

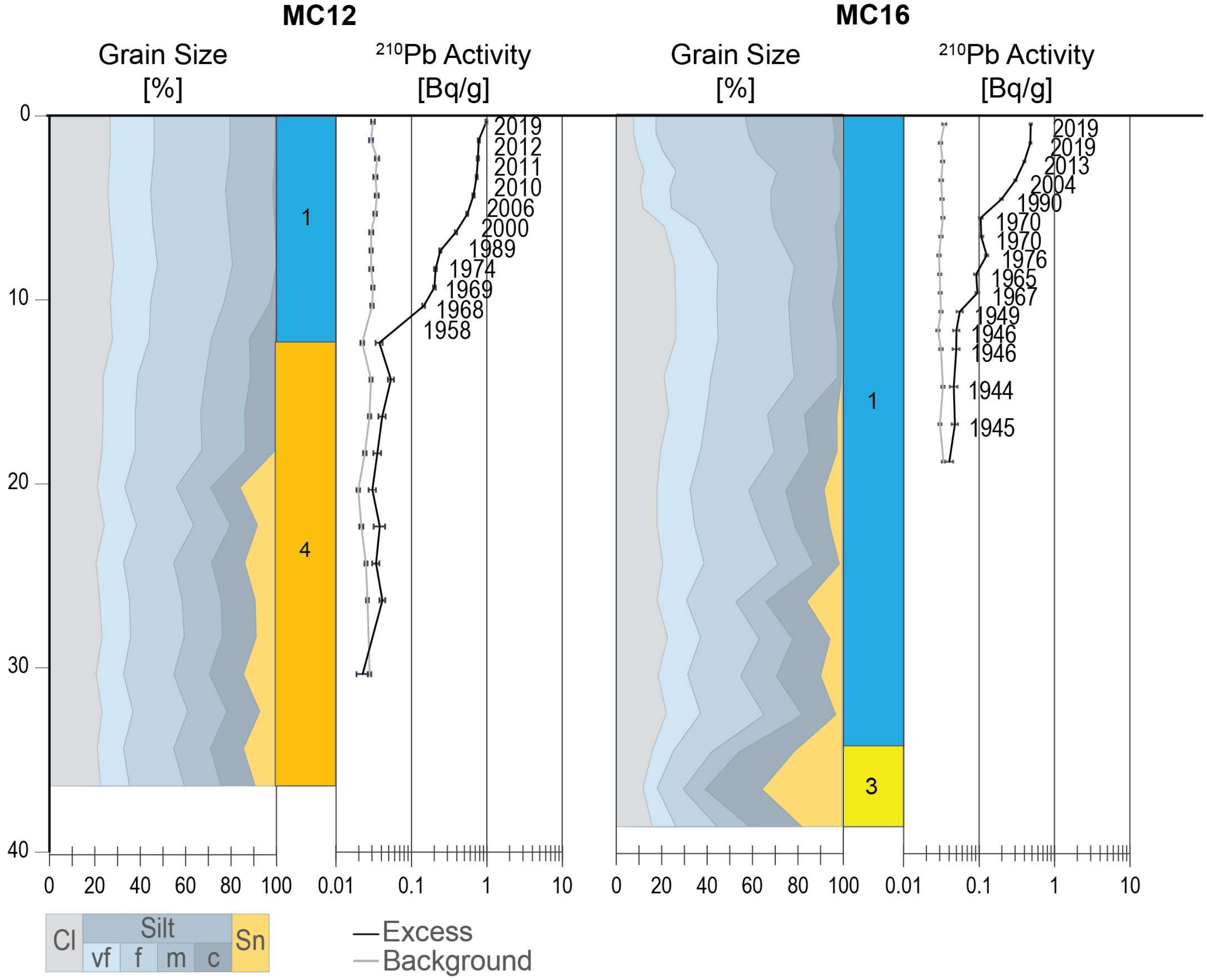

Grain size data, facies assignments, and ^{210}Pb activity for coresMC12 (= site JGC11) and MC16 (= site KC15). Calculated ages (in years C.E.) are given next to the ^{210}Pb profiles. Error bars are shown for individual background and excess ^{210}Pb activity measurements.

Supplementary Information Tables

Table S1. Metadata for cores presented in this study.

Core Number	Corer Type	Recovery (m)	Latitude (° S)	Longitude (° W)	Water Depth (m)
KC04	Kasten	2.5	74.947	106.877	469
JGC11	Jumbo gravity	2.6	75.058	107.229	752
MC12	Megacorer	0.4	75.058	107.229	748
KC13	Kasten	3	74.911	106.953	463
KC15	Kasten	3	74.871	106.333	545
MC16	Megacorer	0.4	74.871	106.333	549
JGC17	Jumbo gravity	1.3	74.887	106.316	507
KC19	Kasten	3	74.863	106.341	704
KC23	Kasten	2.1	75.071	104.228	677

Table S2 Radiocarbon age data and calibrated ages.

Core	Lab Code	Depth Interval [cm]	Dated Material	^{14}C Age [yrs BP ± 1σ]	ΔR	CALIB 8.2 age [yrs BP]	2σ Age Range [yrs BP]
KC04	ETH-105651	92 to 100	Benthic foraminifera	1630 ± 50	843 ± 40	250	50 to 440
KC04	ETH-105652	122 to 130	Benthic foraminifera	1670 ± 60	843 ± 40	290	90 to 480
KC15	ETH-105653	262 to 266	Benthic foraminifera	8550 ± 90	843 ± 40	7990	7740 to 8230
KC15	ETH-105654	292 to 296	Benthic foraminifera	9060 ± 70	843 ± 40	8530	8310 to 8800
KC19	ETH-105655	297.6 to 301	Benthic foraminifera	9740 ± 70	843 ± 40	9400	9140 to 9590

Table S3 Clay mineral provenance data for KC04, KC15 and KC19.

Core	Depth Interval [cm]	Smectite [%]	Illite [%]	Chlorite [%]	Kaolinite [%]
KC04	0-1	22.8	32.5	9.1	35.5
KC04	7-8	19.5	35.0	8.3	37.1
KC04	15-16	21.7	30.4	8.8	39.1
KC04	19-20	23.3	29.7	8.8	38.2
KC04	42-43	30.5	26.2	8.6	34.7
KC04	58-59	26.3	28.5	8.0	37.2
KC04	67-68	33.7	25.8	7.8	32.7
KC04	76-77	30.6	26.6	8.1	34.7
KC04	86-88	24.1	28.5	8.3	39.1
KC04	117-118	32.0	23.7	7.3	36.9
KC04	128-129	34.2	24.5	6.9	34.4
KC04	144-145	29.7	27.1	7.6	35.6
KC04	156-157	31.0	22.1	8.3	38.6
KC04	180-181	28.3	25.9	7.8	38.0
KC04	205-206	23.0	29.4	8.0	39.5
KC04	221-222	33.8	26.2	7.9	32.1
KC04	229-230	23.7	28.9	8.1	39.4
KC04	248-249	23.4	29.2	8.3	39.1
KC15	0-1	17.4	31.3	8.5	42.8
KC15	4-5	20.0	31.1	8.6	40.3
KC15	17-18	19.1	31.7	8.6	40.7
KC15	36-37	19.0	32.4	9.3	39.2
KC15	70-71	19.4	30.7	8.7	41.2
KC15	81-82	18.4	33.2	8.9	39.5
KC15	116-117	19.2	34.2	9.8	36.8
KC15	150-151	21.9	34.2	9.5	34.4
KC15	176-177	21.8	30.6	8.7	39.0
KC15	185-186	22.3	35.1	9.8	32.7
KC15	243-244	21.2	32.9	9.8	36.1
KC15	255-256	16.0	43.2	11.9	28.8
KC15	290-291	18.7	41.3	12.1	27.9
KC19	0	18.6	31.6	8.5	41.2
KC19	8	18.4	32.2	8.7	40.7
KC19	11	17.9	31.1	8.6	42.4
KC19	30	17.4	33.1	9.0	40.6
KC19	42	16.0	35.9	9.0	39.2
KC19	101	15.8	37.1	9.8	37.3
KC19	130	16.4	38.3	9.9	35.5
KC19	150	17.0	35.5	8.4	39.1
KC19	193	17.4	33.2	9.4	39.9
KC19	250	16.0	35.7	10.0	38.2
KC19	274	15.8	42.7	10.7	30.7
KC19	290	16.3	41.6	11.3	30.9
